# Genistein and Procyanidin B2 Reduce Carcinogen-Induced Reactive Oxygen Species and DNA Damage through the Activation of Nrf2/ARE Cell Signaling in Bronchial Epithelial Cells In Vitro

**DOI:** 10.3390/ijms24043676

**Published:** 2023-02-12

**Authors:** Tharindu L. Suraweera, J. P. Jose Merlin, Graham Dellaire, Zhaolin Xu, H. P. Vasantha Rupasinghe

**Affiliations:** 1 Department of Plant, Food, and Environmental Sciences, Faculty of Agriculture, Dalhousie University, Truro, NS B2N 2R8, Canada; 2 Department of Pathology, Faculty of Medicine, Dalhousie University, Halifax, NS B3H 4H7, Canada; 3 QEII Health Sciences Centre, Division of Anatomical Pathology and Cytopathology, Nova Scotia Health Authority, Halifax, NS B3H 1V8, Canada

**Keywords:** dietary flavonoids, carcinogenesis, lung epithelial cells, antioxidant defense system, quercetin

## Abstract

Cancer is one of the leading causes of death worldwide. Chemotherapy and radiation therapy are currently providing the basis for cancer therapies, although both are associated with significant side effects. Thus, cancer prevention through dietary modifications has been receiving growing interest. The potential of selected flavonoids in reducing carcinogen-induced reactive oxygen species (ROS) and DNA damage through the activation of nuclear factor erythroid 2 p45 (NF-E2)-related factor (Nrf2)/antioxidant response element (ARE) pathway was studied in vitro. Dose-dependent effects of pre-incubated flavonoids on pro-carcinogen 4-[(acetoxymethyl)nitrosamino]-1-(3-pyridyl)-1-butanone (NNKAc)-induced ROS and DNA damage in human bronchial epithelial cells were studied in comparison to non-flavonoids. The most effective flavonoids were assessed for the activation of Nrf2/ARE pathway. Genistein, procyanidin B2 (PCB2), and quercetin significantly suppressed the NNKAc-induced ROS and DNA damage. Quercetin significantly upregulated the phosphorylated protein kinase B/Akt. PCB2 significantly upregulated the activation of Nrf2 and Akt through phosphorylation. Genistein and PCB2 significantly upregulated the phospho-Nrf2 nuclear translocation and catalase activity. In summary, genistein and PCB2 reduced the NNKAc-induced ROS and DNA damage through the activation of Nrf2. Further studies are required to understand the role of dietary flavonoids on the regulation of the Nrf2/ARE pathway in relation to carcinogenesis.

## 1. Introduction

According to the World Health Organization (WHO), cancer is the second leading cause of death worldwide after deaths caused by heart diseases [1]. In 2020, nearly 19.3 million new cancer cases and 10 million cancer-related deaths occurred [2]. Among observed cancers, breast, lung, colorectal, and prostate cancers were the most common [2]. Even though there are many therapeutic approaches such as chemotherapy, radiation, immunotherapy, and surgery to overcome cancers, these treatments are known to cause severe side effects [3,4,5]. Even after therapeutic treatments on cancers, the recurrence and less than five-year net survival rates of several cancers such as lung cancer are common among cancer patients [6].

WHO recommends regular consumption of adequate fruits and vegetables to reduce the risk of cancer (1). Phytochemicals present in fruits and vegetables have been shown to reduce the prevalence of cancer in numerous studies [7]. Therefore, dietary phytochemicals have been gaining attention in the prevention of cancers [8]. The reported cancer prevention by dietary phytochemicals, especially (poly)phenols such as flavonoids, stilbenes, curcuminoids, and phenolic acids have been associated with their antioxidant properties [9]. These phytochemicals can mitigate oxidative stress caused by the excessive generation of reactive oxygen species (ROS) [7]. Cellular ROS generation can be induced by both endogenous and exogenous factors [10]. The mitochondrial electron transport chain is the primary endogenous source of ROS [11], and ionization radiation, hypoxia, smoking, xenobiotics, and air pollutants can contribute to cellular ROS production as exogenous stimuli [12,13]. ROS has the potential to damage cellular DNA, which can cause cancer if DNA repair is inefficient or leads to errors [14]. DNA damage can be reduced by dietary antioxidants such as flavonoids by different mechanisms such as scavenging ROS, metal ion chelating, oxidative enzyme inhibition, and providing antioxidant enzyme cofactors, among others [8,15]. Dietary antioxidants can also activate cellular signaling pathways such as the nuclear factor erythroid 2-related factor 2 (Nrf2)/antioxidant response element (ARE) pathway against oxidative stress [7,16].

In general, the Nrf2/ARE pathway is activated by oxidative stress [17,18]. The activation of this pathway plays a significant role in the prevention of DNA damage and possible carcinogenesis by managing oxidative stress via the expression of antioxidant defense enzymes (superoxide dismutase, glutathione peroxidase, and catalase) and phase 2 detoxifying enzymes [17,18]. Upon expression of these proteins, cytoprotection is provided against endogenous and exogenous carcinogenic stimuli through mechanisms such as oxidation of drugs or xenobiotics, conjugation of oxidized metabolites, and transportation of metabolites out of the cellular environment, which restores cellular redox homeostasis [16]. Phytochemicals such as flavonoids can activate the Nrf2/ARE pathway and upregulate the expression of antioxidant and phase 2 detoxifying enzymes even at a stage devoid of oxidative inducers [19]. However, limited studies have been carried out on flavonoids in carcinogen-induced experimental models with respect to the reduction of ROS and DNA damage through the activation of the Nrf2/ARE pathway by the physiologically relevant concentrations in vitro.

In this study, we employed an in vitro model of carcinogen-induced lung damage, in which the normal bronchial epithelial BEAS-2B cells were treated with a known carcinogen in cigarette smoke, 4-[(acetoxymethyl)nitrosamino]-1-(3-pyridyl)-1-butanone (NNKAc) [20]. Furthermore, emphasis was given to studying the activation of the Nrf2/ARE pathway by the most effective flavonoids at physiologically-relevant concentrations in reducing carcinogen-induced ROS and DNA damage in bronchial epithelial cells.

## 2. Results

### 2.1. Effects of Dietary Antioxidants in the Reduction of NNKAc-Induced ROS Generation in BEAS-2B Cells

**Figure 1:**
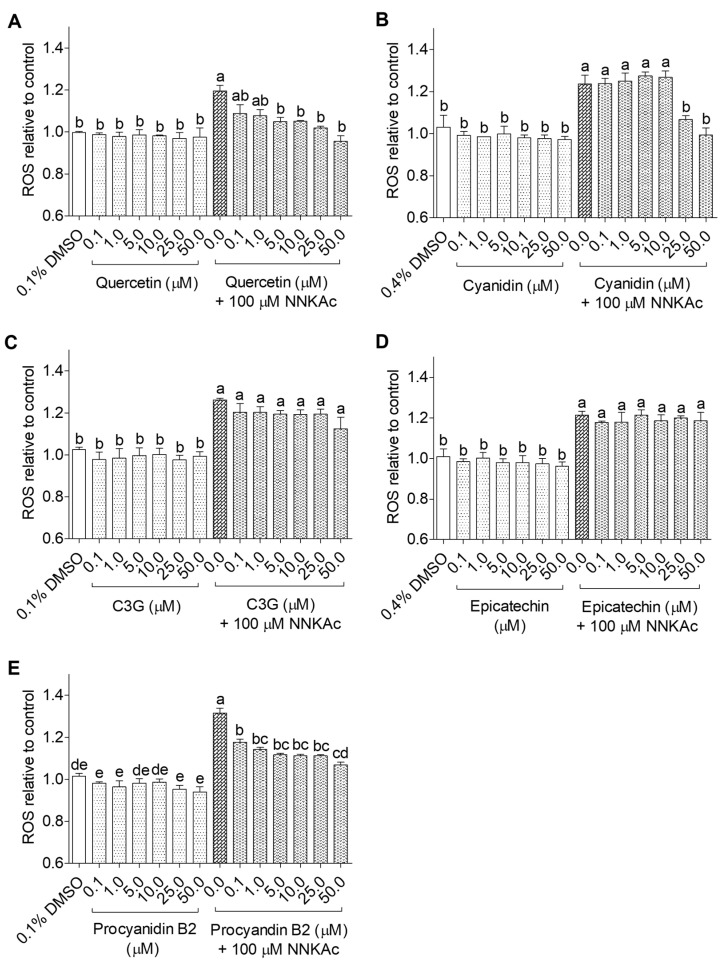
Effect of quercetin (**A**), cyanidin (**B**), cyanidin-3-*O*-glucoside (**C**), epicatechin (**D**), and procyanidin B2 (**E**) on reducing NNKAc-induced ROS in BEAS-2B cells. Cells were pre-treated with concentrations ranging from 0.1 to 50 µM of 3-hydroxy flavonoids for 3 h. Pre-treated cells were exposed to 100 µM NNKAc for another 3 h to induce ROS generation. DMSO (0.1 or 0.4%) was used as the vehicle control. Effects on ROS levels were quantified using DCFDA fluorescence assay. Two independent studies (*n* = 3) were performed, and results were expressed as mean ± standard deviation. Statistical analysis of data was performed by one-way ANOVA and mean comparison was done by Tukey’s mean comparison method (α = 0.05) using Minitab 19 statistical software (LLC, Pennsylvania, USA). Mean values that do not share similar letters in bar graphs are significantly different (*p* < 0.05). Abbreviations: NNKAc: 4-[(acetoxymethyl)nitrosamino]-1-(3-pyridyl)-1-butanone, ROS: reactive oxygen species, DCFDA: 2′ 7′-dichlorofluorescein diacetate, DMSO: dimethylsulfoxide, C3G: cyanidin-3-*O*-glucoside.

**Figure 2:**
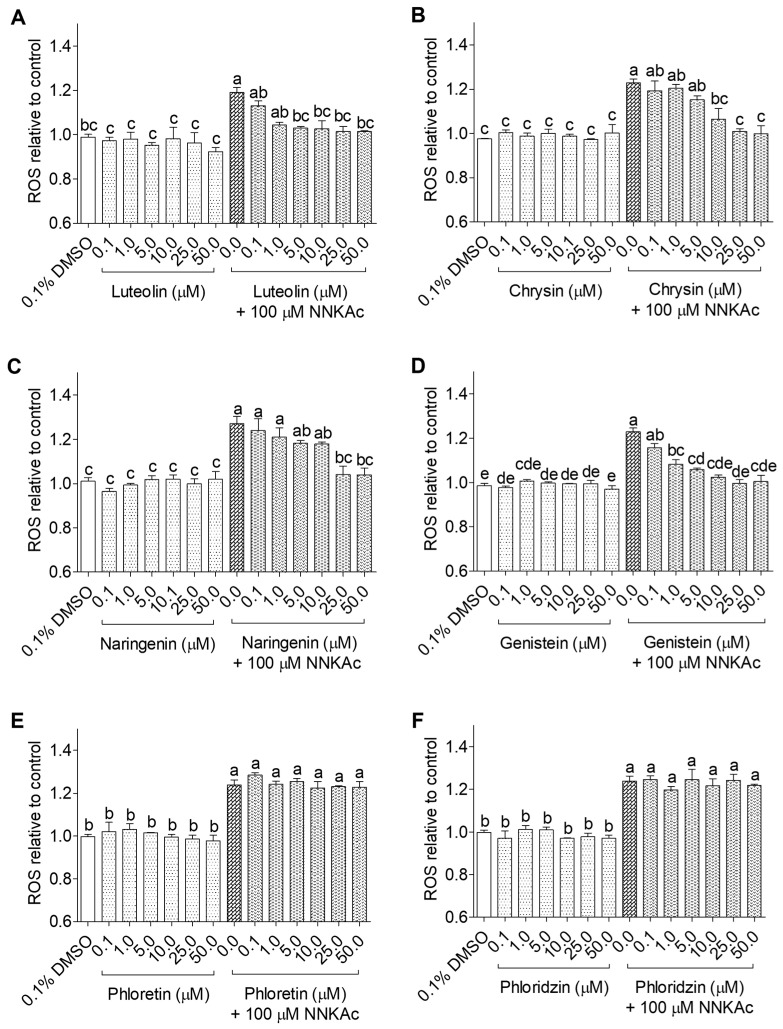
Effect of luteolin (**A**), chrysin (**B**), naringenin (**C**), genistein (**D**), phloretin (**E**), and phloridzin (**F**) on reducing NNKAc-induced ROS in BEAS-2B cells. Cells were pre-treated with concentrations ranging from 0.1 to 50 µM of selected 3-deoxy flavonoids and chalcones for 3 h. Pre-treated cells were exposed to 100 µM NNKAc for another 3 h to induce ROS generation. DMSO (0.1%) was used as the vehicle control. Effects on ROS levels were quantified using DCFDA fluorescence assay. Two independent studies (*n* = 3) were performed, and results were expressed as mean ± standard deviation. Statistical analysis of data was performed by one-way ANOVA and mean comparison was done by Tukey’s mean comparison method (α = 0.05) using Minitab 19 statistical software (LLC, Pennsylvania, USA). Mean values that do not share similar letters in bar graphs are significantly different (*p* < 0.05). Abbreviations: NNKAc: 4-[(acetoxymethyl)nitrosamino]-1-(3-pyridyl)-1-butanone, DCFDA: 2′ 7′-dichlorofluorescein diacetate, ROS: reactive oxygen species, DMSO: dimethylsulfoxide.

The dose-dependent effects of selected 25 dietary antioxidants on NNKAc-induced intracellular ROS generation in BEAS-2B cells were studied using the DCFDA assay. NNKAc-treated BEAS-2B cells showed significantly increased (*p* < 0.05) ROS levels by 20–30% compared to DMSO control. BEAS-2B cells treated with different concentrations (0.1–50 µM) of dietary antioxidants alone did not influence the ROS level (*p* > 0.05) when compared to the DMSO control. Among tested flavonoids, pre-exposure of BEAS-2B cells to 3-hydroxy flavonoids (Figure 1) such as quercetin (5–50 µM), cyanidin (25–50 µM), and procyanidin B2 (0.1–50 µM) and 3-deoxy flavonoids (Figure 2) such as luteolin (5–50 µM), chrysin (10–50 µM), naringenin (25–50 µM), and genistein (1–50 µM) showed significant reductions (*p* < 0.05) in NNKAc-induced ROS levels. However, epicatechin, C3G, phloretin, and phloridzin did not prevent NNKAc-induced ROS generation.

**Figure 3:**
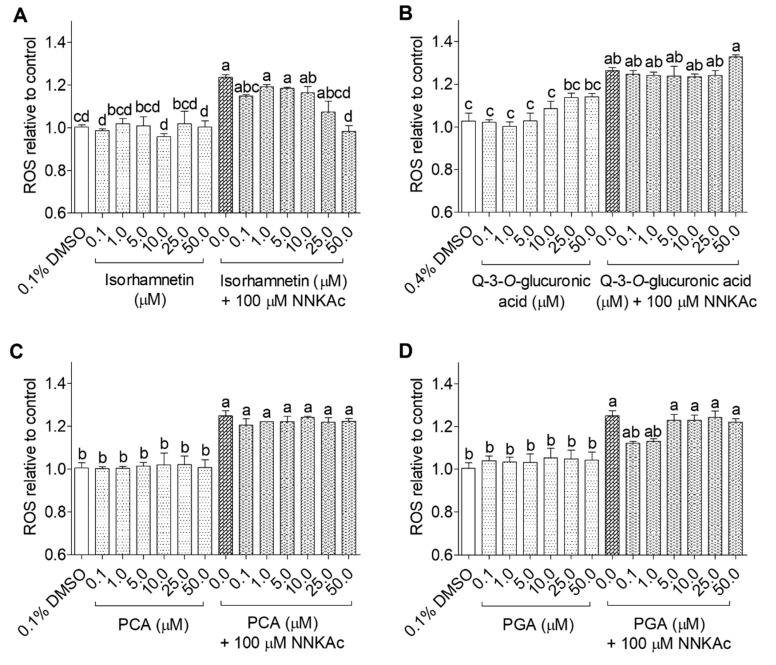
Effect of isorhamnetin (**A**), quercetin-3-*O*-glucuronic acid (**B**), protocatechuic acid (**C**), and phloroglucinaldehyde (**D**) on reducing NNKAc-induced ROS in BEAS-2B cells. Cells were pre-treated with concentrations ranging from 0.1 to 50 µM of selected (poly)phenol metabolites for 3 h. Pre-treated cells were exposed to 100 µM NNKAc for another 3 h to induce ROS generation. DMSO (0.1% or 0.4%) was used as the vehicle control. Effects on ROS levels were quantified using DCFDA fluorescence assay. Two independent studies (*n* = 3) were performed, and results were expressed as mean ± standard deviation. Statistical analysis of data was performed by one-way ANOVA and mean comparison was done by Tukey’s mean comparison method (α = 0.05) using Minitab 19 statistical software (LLC, Pennsylvania, USA). Mean values that do not share similar letters in bar graphs are significantly different (*p* < 0.05). Abbreviations: NNKAc: 4-[(Acetoxymethyl)nitrosamino]-1-(3-pyridyl)-1-butanone, DCFDA: 2′ 7′-dichlorofluorescein diacetate, ROS: reactive oxygen species, DMSO: dimethylsulfoxide, Q: quercetin, PCA: protocatechuic acid, PGA: phloroglucinaldehyde.

**Figure 4:**
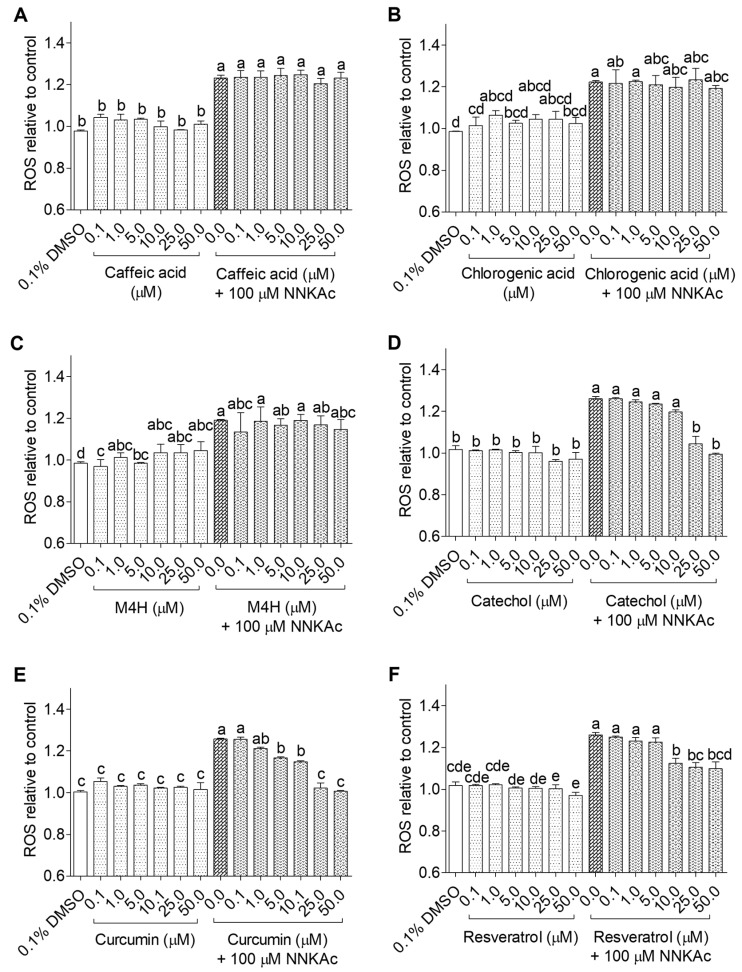
Effect of caffeic acid (**A**), chlorogenic acid (**B**), methyl 4-hydroxybenzoate (**C**), catechol (**D**), curcumin (**E**), and resveratrol (**F**) on reducing NNKAc-induced ROS in BEAS-2B cells. Cells were pre-treated with concentrations ranging from 0.1 to 50 µM of selected phenolic acids and other (poly)phenols for 3 h. Pre-treated cells were exposed to 100 µM NNKAc for another 3 h to induce ROS generation. DMSO (0.1%) was used as the vehicle control. Effects on ROS levels were quantified using DCFDA fluorescence assay. Two independent studies (*n* = 3) were performed, and results were expressed as mean ± standard deviation. Statistical analysis of data was performed by one-way ANOVA and mean comparison was done by Tukey’s mean comparison method (α = 0.05) using Minitab 19 statistical software (LLC, Pennsylvania, USA). Mean values that do not share similar letters in bar graphs are significantly different (*p* < 0.05). Abbreviations: M4H: methyl 4-hydroxybenzoate NNKAc: 4-[(acetoxymethyl)nitrosamino]-1-(3-pyridyl)-1-butanone, DCFDA: 2′ 7′-dichlorofluorescein diacetate, ROS: reactive oxygen species, DMSO: dimethylsulfoxide.

**Figure 5:**
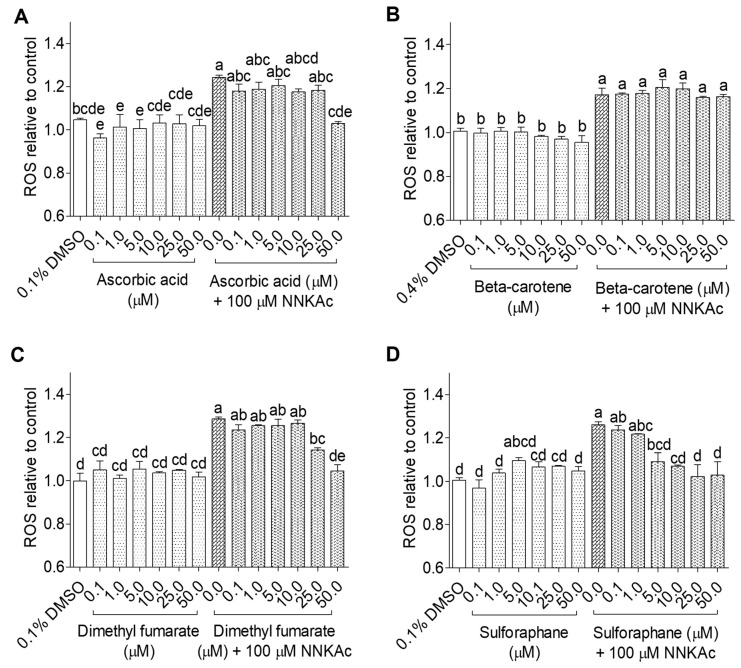
Effect of ascorbic acid (**A**), beta-carotene (**B**), dimethyl fumarate (**C**), and sulforaphane (**D**) on reducing NNKAc-induced ROS in BEAS-2B cells. Cells were pre-treated with concentrations ranging from 0.1 to 50 µM of selected non-phenolic compounds for 3 h. Pre-treated cells were exposed to 100 µM NNKAc for another 3 h to induce ROS generation. DMSO (0.1% or 0.4%) was used as the vehicle control. Effects on ROS levels were quantified using DCFDA fluorescence assay. Two independent studies (*n* = 3) were performed, and results were expressed as mean ± standard deviation. Statistical analysis of data was performed by one-way ANOVA and mean comparison was done by Tukey’s mean comparison method (α = 0.05) using Minitab 19 statistical software (LLC, Pennsylvania, USA). Mean values that do not share similar letters in bar graphs are significantly different (*p* < 0.05). Abbreviations: NNKAc: 4-[(Acetoxymethyl)nitrosamino]-1-(3-pyridyl)-1-butanone, DCFDA: 2′ 7′-dichlorofluorescein diacetate, ROS: reactive oxygen species, DMSO: dimethyl sulfoxide.

Among the tested flavonoid metabolites, isorhamnetin at 50 μM significantly reduced (*p* < 0.05) ROS levels induced by NNKAc in BEAS-2B cells (Figure 3). BEAS-2B cells pretreated with curcumin (5–50 µM), resveratrol (10–50 µM), and catechol (25–50 µM) showed significantly reduced (*p* < 0.05) ROS levels in NNKAc-treated cells (Figure 4). However, BEAS-2B cells pretreated with phenolic acids (i.e., caffeic acid, chlorogenic acid) and methyl 4-hydroxybenzoate did not reduce (*p* > 0.05) NNKAc-induced ROS levels (Figure 4). Furthermore, pretreated BEAS-2B cells with non-phenolic compounds such as ascorbic acid (50 µM), sulforaphane (5–50 µM), and dimethyl fumarate (25–50 µM) also showed significant reductions (*p* < 0.05) in NNKAc-induced ROS levels at different concentrations (Figure 5). However, pre-exposure with beta-carotene was not effective in the reduction (*p* > 0.05) in NNKAc-induced ROS in BEAS-2B cells (Figure 5).

### 2.2. Effects of Dietary Antioxidants in the Reduction of NNKAc-Induced DNA Damage in BEAS-2B Cells

To assess cytoprotective and genoprotective effects against NNKAc-challenged BEAS-2B cells, the dose-dependent effects of 12 selected compounds. The 12 compounds were selected based on their ability to reduce the NNKAc-induced ROS generation in BEAS-2B cells at concentrations equal or less than 25 µM. The selected compounds include: 3-hydroxy (quercetin, cyanidin, and procyanidin B2) and 3-deoxy (luteolin, chrysin, naringenin, and genistein) flavonoids, simple (poly)phenols (catechol), stilbenes (resveratrol), curcuminoids (curcumin) and non-phenolic compounds (DMF and sulforaphane).

#### 2.2.1. Effects of Dietary Antioxidants on BEAS-2B Cell Viability

The effects of test compounds on cell viability under experimental conditions were studied using the MTS assay (Supplementary Figures S1 and S2). The viability of BEAS-2B cells was reduced by 10–20% due to the exposure of 100 µM NNKAc but the reduction was not significant (*p* > 0.05) when compared to the DMSO control. The test compounds at concentrations of 0.1–25 μM did not impact (*p* > 0.05) on the viability of BEAS-2B cells when compared to the DMSO control.

#### 2.2.2. Effects of Dietary Antioxidants on NNKAc-Induced DNA Damage in BEAS-2B Cells

**Figure 6:**
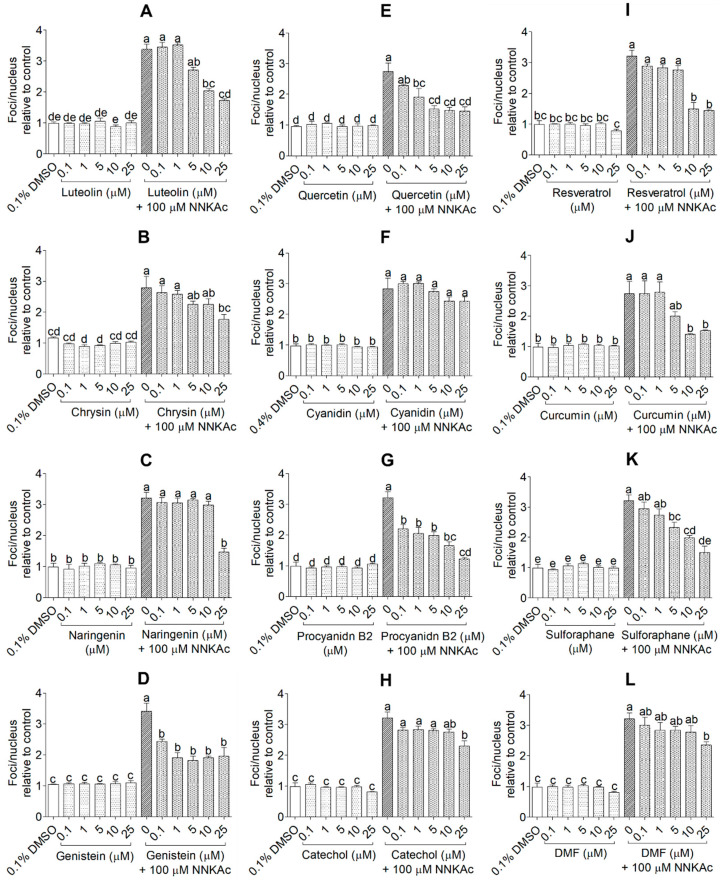
Effect of luteolin (**A**), chrysin (**B**), naringenin (**C**), genistein (**D**), quercetin (**E**), cyanidin (**F**), procyanidin B2 (**G**), catechol (**H**), resveratrol (**I**), curcumin (**J**), sulforaphane (**K**), and DMF (**L**) on NNKAc-induced DNA damage in BEAS-2B cells measured by γ-H2AX immunofluorescence assay. Cells were pre-treated with concentrations ranging from 0.1 to 25 µM of selected compounds for 3 h. Pre-treated cells were exposed to 100 µM NNKAc for another 3 h. DMSO (0.1%) was used as the vehicle control. Specific antibodies were used to label the phosphorylated histone γ-H2AX foci (S139) and DAPI was used to stain the nucleus. The foci/nucleus ratio was quantified using at least 50 nuclei per treatment. Nuclei were imaged by fluorescence microscopy (×100 magnification). The foci per nuclei were counted by ImageJ software (Version 1.53k, National Institute of Mental Health, Bethesda, MD, USA). Three independent studies were performed, and results were expressed as mean ± standard deviation. Statistical analysis of data was performed by one-way ANOVA and mean comparison was done by Tukey’s mean comparison method (α = 0.05) using Minitab 19 statistical software (LLC, Pennsylvania, USA). Mean values that do not share similar letters in bar graphs are significantly different (*p* < 0.05). Abbreviations: NNKAc: 4-[(Acetoxymethyl)nitrosamino]-1-(3-pyridyl)-1-butanone, DMF: dimethyl fumarate, DMSO: dimethyl sulfoxide.

**Figure 7:**
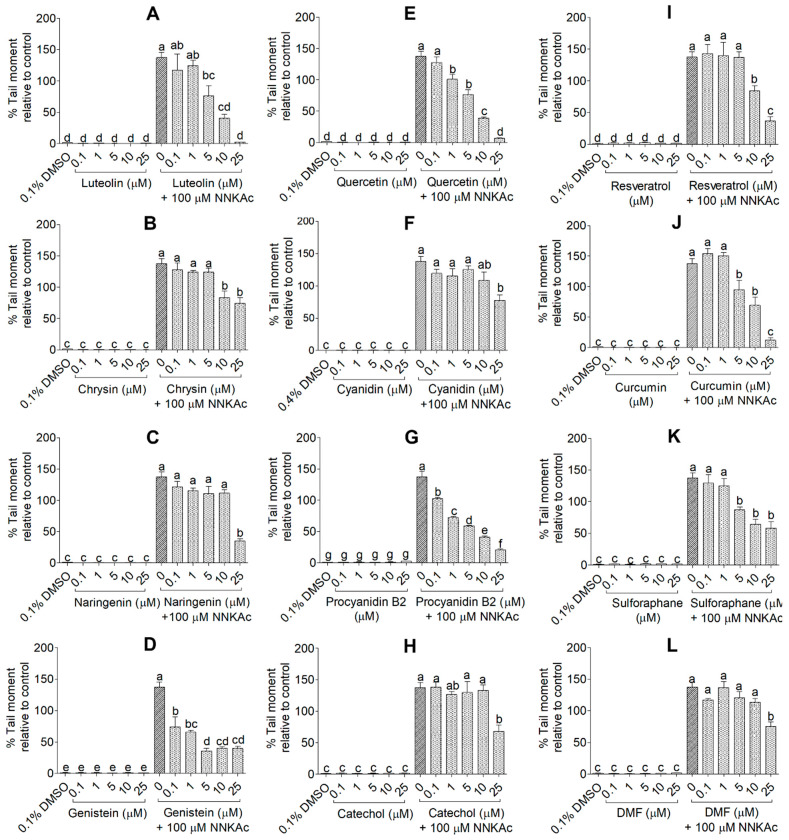
Effect of luteolin (**A**), chrysin (**B**), naringenin (**C**), genistein (**D**), quercetin (**E**), cyanidin (**F**), procyanidin B2 (**G**), catechol (**H**), resveratrol (**I**), curcumin (**J**), sulforaphane (**K**), and DMF (**L**) on NNKAc-induced DNA damage in BEAS-2B cells measured by comet assay. Cells were pre-treated with concentrations ranging from 0.1 to 25 µM of selected compounds for 3 h. Pre-treated cells were exposed to 100 µM NNKAc for another 3 h. DMSO (0.1%) was used as the vehicle control. Comets were imaged by fluorescence microscopy (×100 magnification). Percentage tail moment using at least 30 comets per each treatment was calculated by using the OpenComet plugin in ImageJ software (Version 1.53k, National Institute of Mental Health, Bethesda, MD, USA). Three independent studies were performed, and results were expressed as mean ± standard deviation. Statistical analysis of data was performed by one-way ANOVA and mean comparison was done by Tukey’s mean comparison method (α = 0.05) using Minitab 19 statistical software (LLC, Pennsylvania, USA). Mean values that do not share similar letters in bar graphs are significantly different (*p* < 0.05). Abbreviations: NNKAc: 4-[(acetoxymethyl)nitrosamino]-1-(3-pyridyl)-1-butanone, DMF: dimethyl fumarate, and DMSO: dimethyl sulfoxide.

**Figure 8:**
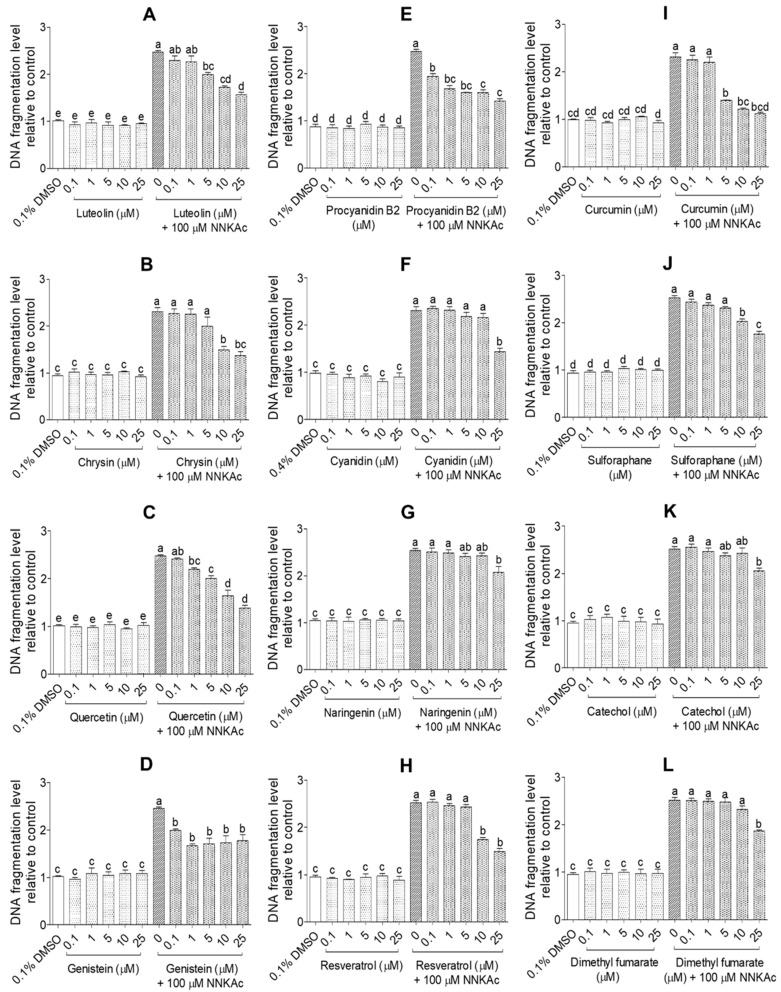
Effect of luteolin (**A**), chrysin (**B**), quercetin (**C**), genistein (**D**), procyanidin B2 (**E**), cyanidin (**F**), naringenin (**G**), resveratrol (**H**), curcumin (**I**), sulforaphane (**J**), catechol (**K**), and DMF (**L**) on DNA fragmentation level against NNKAc-induced DNA damage in BEAS-2B cells. Cells were pre-treated with concentrations ranging from 0.1 to 25 µM of selected compounds for 3 h. Pre-treated cells were exposed to 100 µM NNKAc for another 3 h. DMSO (0.1% or 0.4%) was used as the vehicle control. Effects on the level of DNA fragmentation were quantified using DNA fragmentation ELISA assay. Three independent studies were performed, and results were expressed as mean ± standard deviation. Statistical analysis of data was performed by one-way ANOVA and mean comparison was done by Tukey’s mean comparison method (α = 0.05) using Minitab 19 statistical software (LLC, Pennsylvania, USA). Mean values that do not share similar letters in bar graphs are significantly different (*p* < 0.05). Abbreviations: NNKAc: 4-[(acetoxymethyl)nitrosamino]-1-(3-pyridyl)-1-butanone, DMF: dimethyl fumarate, and DMSO: dimethyl sulfoxide.

The protective effects of selected test compounds on NNKAc-induced DNA damage in BEAS-2B cells were studied using three assays. Effects on DNA double-strand breaks (DSBs) induced by NNKAc in BEAS-2B cells were evaluated by quantifying γ-H2AX foci per nucleus using the immunofluorescence assay (HIA; Figure 6 and Supplementary Figures S3–S5). Percentage tail moment of single BEAS-2B cells from comet assay was used to study the protective effects of tested compounds on both DNA single-strand breaks (SSBs) and DNA DSBs induced by NNKAc in BEAS-2B cells (CA; Figure 7 and Supplementary Figures S6–S8). For DNA fragmentation by ELISA assay, absorbance at 450 nm was recorded to measure the level of DNA fragmentation by spectrophotometry (DFEA; Figure 8).

BEAS-2B cells-treated with NNKAc showed significantly higher γ-H2AX foci per nucleus, percentage DNA tail moment, and DNA fragmentation levels (*p* < 0.05) compared to DMSO control (Figure 6, Figure 7 and Figure 8). BEAS-2B cells treated with different concentrations (0.1–25 μM) of all 12 selected dietary antioxidants without NNKAc challenge showed no significant DNA damage (*p* > 0.05) in all three experiments compared to DMSO control. BEAS-2B cells pre-treated with quercetin (1–25 μM), procyanidin B2 (0.1–25 μM), and genistein (0.1–25 μM) showed significant (*p* < 0.05) reductions in γ-H2AX foci per nucleus, percentage DNA tail moment, and DNA fragmentation levels in BEAS-2B cells against NNKAc challenge at both low and high concentrations (Figure 6, Figure 7 and Figure 8). Pre-treatment with luteolin (HIA: 10–25 µM, CA: 5–25 µM, and DFEA: 5–25 µM), chrysin (HIA: 25 µM, CA: 10–25 µM and DFEA: 10–25 µM), naringenin (HIA: 25 µM, CA: 25 µM and DFEA: 25 µM) and cyanidin (CA: 25 µM and DFEA: 25 µM) significantly reduced (*p* < 0.05) NNKAc-induced DNA damage in BEAS-2B cells at comparatively higher concentrations than genistein, procyanidin B2, and quercetin. Curcumin (DFEA: 5–25 µM, HIA: 10–25 µM, and CA:5–25 µM), catechol (DFEA: 25 µM, HIA: 25 µM, and CA: 25 µM), resveratrol (DFEA: 10–25 µM, HIA: 10–25 µM, and CA: 10–25 µM), sulforaphane (DFEA: 10–25 µM, HIA: 5–25 µM, and CA: 5–25 µM), and dimethyl fumarate (DFEA: 25 µM, HIA: 25 µM, and CA: 25 µM) also significantly (*p* < 0.05) reduced NNKAc-induced DNA damage in BEAS-2B cells compared to BEAS-2B cells treated with NNKAc at relatively higher concentrations.

### 2.3. Effects of Quercetin, Genistein, and Procyanidin B2 on Nrf2/ARE Signaling Pathway in BEAS-2B Cells

To assess the effects on the Nrf2/ARE pathway in NNKAc-challenged and normal BEAS-2B cells, the dose-dependent (1 and 25 µM) effects of the most effective flavonoids (quercetin, genistein, and procyanidin B2) on phosphorylation of Akt and Nrf2 in BEAS-2B cells were studied.

#### 2.3.1. Effect of Quercetin, Genistein, and Procyanidin B2 on the Phosphorylation of Akt and Nrf2 in BEAS-2B Cells

**Figure 9:**
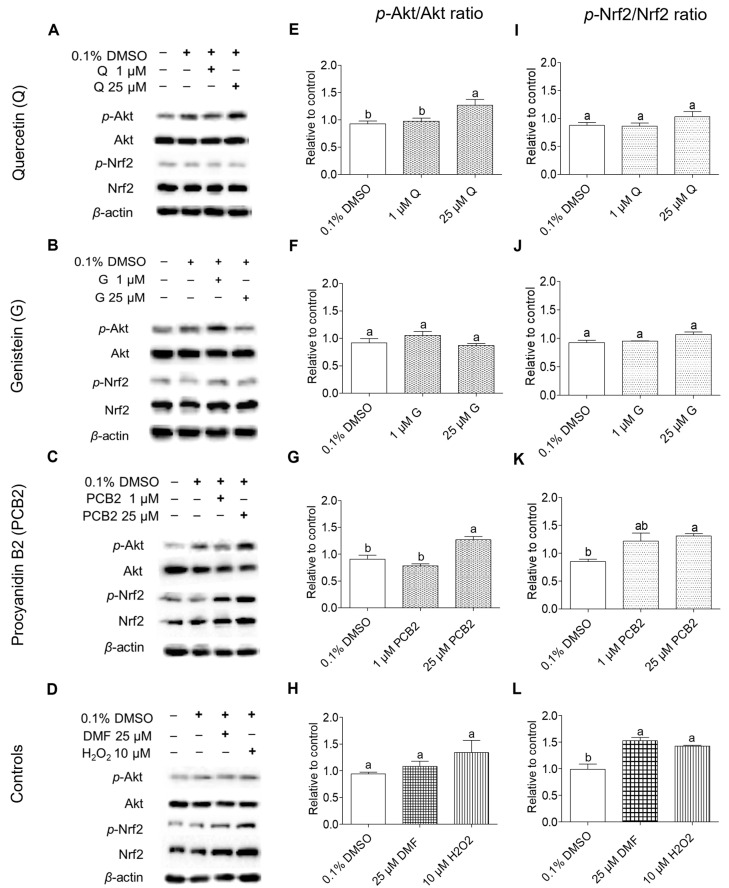
Effect of quercetin (**A**), genistein (**B**), procyanidin B2 (**C**), and controls (**D**) on phosphorylation of Nrf2 and Akt proteins in BEAS-2B cells. Cells were treated with 1 µM and 25 µM of quercetin, genistein, or procyanidin B2 for 3 h. DMF (25 µM) and H2O2 (10 µM) were used as the positive controls and 0.1 % DMSO was used as the vehicle control. The relative expression of p-Akt (S473) (**E**–**H**) and p-Nrf2 (S40) (**I**–**L**) protein levels to their respective total protein expressions were quantified using western blot analysis. At least three independent studies were performed, and results were expressed as mean ± standard deviation. Statistical analysis of data was performed by one-way ANOVA and mean comparison was done by Tukey’s mean comparison method (α = 0.05) using Minitab 19 statistical software ( LLC, Pennsylvania, USA). Mean values that do not share similar letters in bar graphs are significantly different (*p* < 0.05). Abbreviations: NNKAc: 4-[(acetoxymethyl)nitrosamino]-1-(3-pyridyl)-1-butanone, DMF: dimethyl fumarate DMSO: dimethyl sulfoxide, H_2_O_2_: hydrogen peroxide, G: genistein, Q: quercetin, PCB2: procyanidin B2.

Western blot analysis was used to assess the effects of quercetin, genistein, and procyanidin B2 on the activation of Nrf2 and its upstream kinase Akt through phosphorylation in BEAS-2B cells (Figure 9). In addition, two positive controls, DMF and hydrogen peroxide, were tested for their effects on Akt and Nrf2 phosphorylation. However, a significant increase (*p* < 0.05) compared to the DMSO control was observed only with the p-Nrf2/Nrf2 ratio but not with the p-Akt/Akt ratio after treatment of BEAS-2B cells with DMF or hydrogen peroxide.

BEAS-2B cells treated with 25 μM quercetin or procyanidin B2 showed a significant increase in the p-Akt/Akt ratio. Genistein treatment showed an increase in the p-Akt/Akt ratio in BEAS-2B cells at the lowest concentration (1 μM) tested, but the observed levels were not significantly different (*p* > 0.05) from the DMSO control. Exposure of BEAS-2B cells only to procyanidin B2 but not quercetin and genistein increased the p-Nrf2/Nrf2 ratio at tested both concentrations (1 and 25 μM). However, a significant increase (*p* < 0.05) in the p-Nrf2/Nrf2 ratio compared to the DMSO control was only observed with procyanidin B2 treatment at its highest tested concentration (25 μM).

#### 2.3.2. Effect of Quercetin, Genistein, and Procyanidin B2 on p-Nrf2 Nuclear Translocation in BEAS-2B Cells

**Figure 10:**
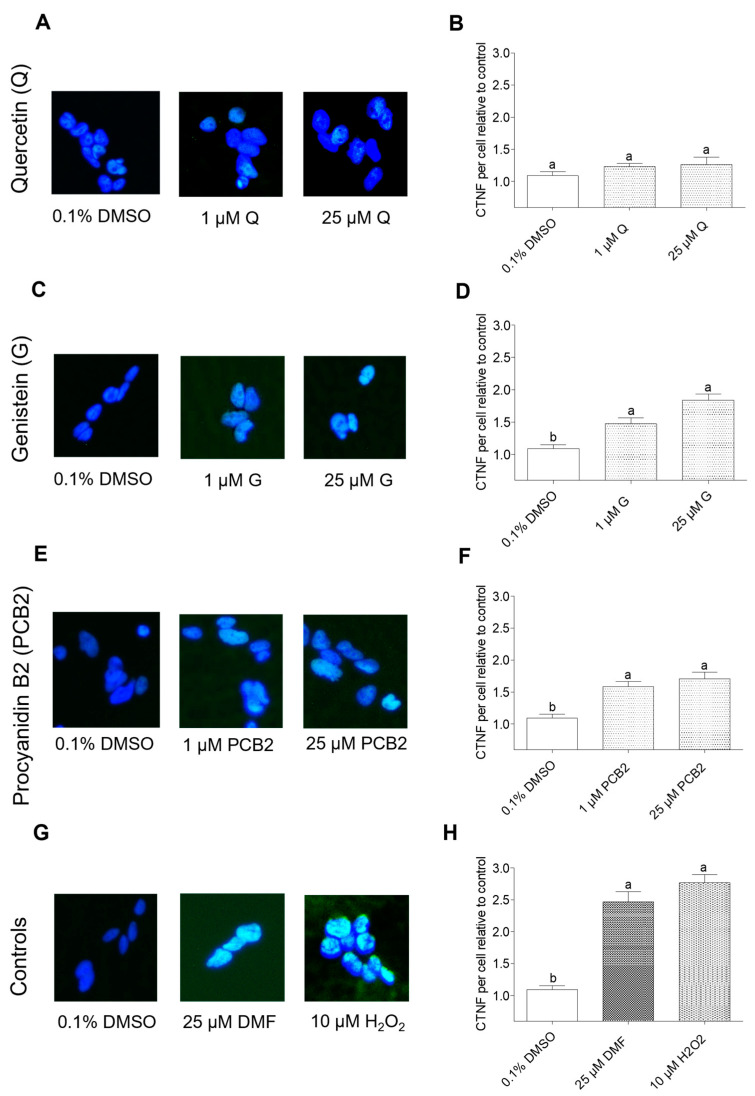
Effect of quercetin (**A**,**B**), genistein (**C**,**D**), procyanidin B2 (**E**,**F**) and controls (**G**,**H**) on p-Nrf2 nuclear translocation in BEAS-2B cells measured by immunofluorescence assay. Cells were treated with 1 µM and 25 µM of quercetin, genistein, or procyanidin B2 for 3 h. DMF (25 µM) and H_2_O_2_ (10 µM) were used as the positive controls, and 0.1% DMSO was used as the vehicle control. Phosphorylated Nrf2 (Ser40) (p-Nrf2) was labeled with specific antibodies and nuclei were stained with DAPI. Nuclei were imaged by fluorescence microscopy (×100 magnification). The corrected nuclear fluorescence (CTNF) levels per nucleus were quantified using at least 30 nuclei per treatment to measure the level of p-Nrf2 nuclear translocation by immunofluorescence analysis. CTNF per nucleus was measured by ImageJ software (Version 1.53k, National Institute of Mental Health, Bethesda, MD, USA). Three independent studies were performed, and results were expressed as mean ± standard deviation. Statistical analysis of data was performed by one-way ANOVA and mean comparison was done by Tukey’s mean comparison method (α = 0.05) using Minitab 19 statistical software (LLC, Pennsylvania, USA). Mean values that do not share similar letters in bar graphs are significantly different (*p* < 0.05). Abbreviations: NNKAc: 4-[(acetoxymethyl)nitrosamino]-1-(3-pyridyl)-1-butanone, DMF: dimethyl fumarate DMSO: dimethyl sulfoxide, H_2_O_2_: hydrogen peroxide, Q: quercetin, G: genistein, PCB2: procyanidin B2.

The immunofluorescence assay was used to assess the effects of quercetin, genistein, and procyanidin B2 on the nuclear translocation of p-Nrf2 in BEAS-2B cells (Figure 10). BEAS-2B cells treated with DMF, or hydrogen peroxide showed significantly (*p* < 0.05) higher levels of p-Nrf2 nuclear translocation compared to DMSO control. BEAS-2B cells treated with quercetin, genistein, or procyanidin B2 showed a dose-dependent increase in nuclear p-Nrf2 levels. However, a significant (*p* < 0.05) increase in nuclear p-Nrf2 levels was observed only with genistein and procyanidin B2 treatments at all tested concentrations.

#### 2.3.3. Effect of Quercetin, Genistein, and Procyanidin B2 on Antioxidant Enzyme Activities in BEAS-2B Cells

**Figure 11:**
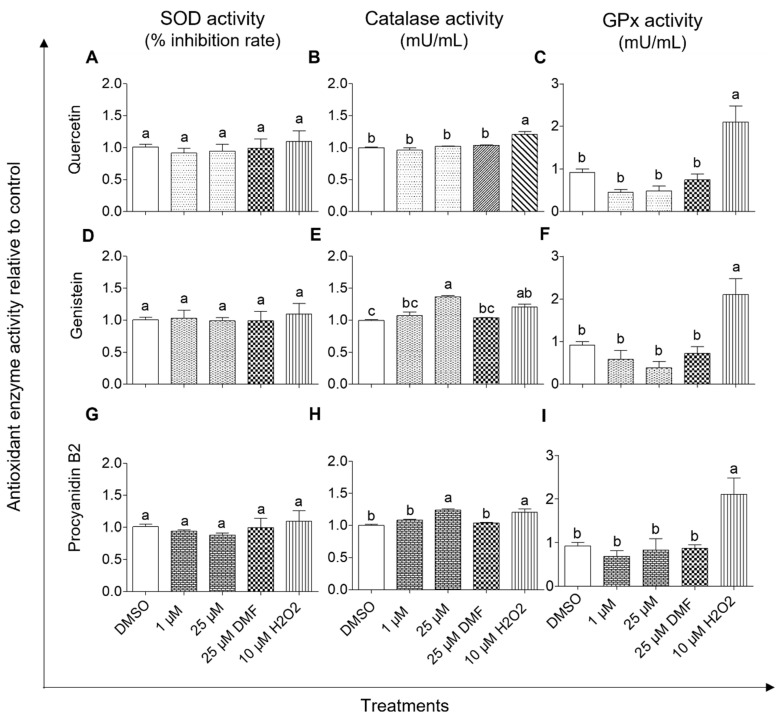
Effect of quercetin (**A**–**C**), genistein (**D**–**F**), and procyanidin B2 (**G**–**I**) on antioxidant enzyme (superoxide dismutase, catalase, and glutathione peroxidase) activities in BEAS-2B cells. Cells were treated with 1 µM or 25 µM of quercetin, genistein, or procyanidin B2 for 3 h. DMF (25 µM) and H_2_O_2_ (10 µM) were used as the positive controls, and 0.1% DMSO was used as the vehicle control. Effects of tested compounds on the activity of SOD, catalase, and GPx were assessed. Three independent studies (each done in duplicate) were performed, and results were expressed as mean ± standard deviation. Statistical analysis of data was performed by one-way ANOVA and mean comparison was done by Tukey’s mean comparison method (α = 0.05) using Minitab 19 statistical software (LLC, Pennsylvania, USA). Mean values that do not share similar letters (i.e., (a–c)) in bar graphs are significantly different (*p* < 0.05). Abbreviations: DMF: dimethyl fumarate DMSO: dimethyl sulfoxide, H_2_O_2_: hydrogen peroxide, GPx: glutathione peroxidase, SOD: superoxide dismutase.

Effects of quercetin, genistein, or procyanidin B2 on antioxidant enzyme activities in BEAS-2B cells were evaluated in terms of SOD (percentage inhibition of superoxide radical), catalase (mU/mL), and GPx (mU/mL) activities (Figure 11). BEAS-2B cells treated with hydrogen peroxide showed a significant (*p* < 0.05) increase in both catalase and GPx activities but not SOD activity compared to DMSO control. Furthermore, no significant changes (*p* > 0.05) were observed in the antioxidant enzyme activities of BEAS-2B cells treated with DMF compared to the DMSO control. Interestingly, BEAS-2B cells treated with 25 μM genistein or 25 μM procyanidin B2 showed a significant increase (*p* < 0.05) in the activity of catalase when compared with DMSO control. Also, no significant changes (*p* > 0.05) were observed in the SOD and GPx activities of BEAS-2B cells treated with quercetin, genistein, or procyanidin B2, even though the GPx activities were lower than those of the DMSO control.

## 3. Discussion

Dietary antioxidants have received increasing interest among scientists, manufacturers, and consumers due to their potential health benefits against many non-communicable diseases. Over the years, dietary (poly)phenols, particularly flavonoids, have been widely studied and reviewed for their physiological benefits, including their role as antioxidants in cancer chemopreventive agents [19,20,21,22,23,24,25,26]. In this study, we investigated the efficacy of selected flavonoids in comparison to flavonoid metabolites, phenolic acids, simple (poly)phenols, stilbenes, curcuminoids, and non-phenolic antioxidants in reducing carcinogen-induced DNA damage in normal lung epithelial BEAS-2B cells. In addition, we also investigated whether the DNA damage reductions observed by the three most effective flavonoids are due to the activation of the Nrf2/ARE pathway.

The in vitro carcinogen-induced human normal bronchial epithelial BEAS-2B cell model [20] was used in this study. Cultured BEAS-2B cells pre-treated with dietary antioxidants were exposed to NNKAc to induce DNA damage. NNKAc mimics the carcinogenic effects of NNK, the most toxic nicotine-derived carcinogen present in tobacco smoke [27]. For the activation and metabolism of NNK, the activities of CYP enzymes are required but the CYP activity in BEAS-2B cells is low [28]. However, NNKAc is activated in a cellular environment by esterase enzymes and CYP activities are not required [29]. Therefore, to induce cyto- and geno-toxicity in BEAS-2B cells, NNKAc has been successfully used in numerous studies [20,30,31].

ROS generation is one of the factors contributing to DNA damage in normal healthy cells [31]. Pretreatment of BEAS-2B cells with flavonoids such as quercetin, cyanidin, procyanidin B2, luteolin, chrysin, naringenin, or genistein except for epicatechin, C3G, phloretin, and phloridzin effectively reduced the NNKAc-induced ROS in BEAS-2B cells in a dose-dependent manner. Among tested antioxidants, only quercetin, luteolin, genistein, and procyanidin B2 were able to reduce the NNKAc-induced ROS at physiologically relevant concentrations [32,33,34,35]. In contrast, tested phenolic acids were not effective in reducing NNKAc-induced ROS levels in BEAS-2B cells. Compared to flavonoids, phenolic acids are weak radical scavengers [36], mainly due to the lower number of hydroxyl groups present in their structures [36]. Catechol, vitamin C and DMF (US FDA-approved Nrf2 activator to treat relapsing forms of multiple sclerosis) reduced the NNKAc-induced ROS levels in BEAS-2B cells at 25 and/or 50 µM. Interestingly, curcumin, resveratrol, and sulforaphane showed comparable effectiveness in reducing NNKAc-induced ROS levels in BEAS-2B cells. However, these results were observed at concentrations higher than physiologically relevant concentrations [37,38,39].

The protective effects of selected flavonoids (quercetin, luteolin, chrysin, naringenin, genistein, cyanidin, and procyanidin B2), DMF, curcumin, sulforaphane, resveratrol, and catechol in reducing NNKAc-induced DNA damage in BEAS-2B cells was studied. DNA damage was assessed using γ-H2AX immunofluorescence, alkaline comet, and DNA fragmentation ELISA assays since altered genetic stability is considered an early event in carcinogenesis [40]. NNKAc cellular metabolism causes DNA damage, which can be characterized by DNA adduct formation and strand breaks [20,29,30,31]. In this study, DNA damage in BEAS-2B cells was induced by NNKAc (100 µM) as observed by γ-H2AX immunofluorescence, comet, and DNA fragmentations-ELISA assays [20,30,31]. Post-translational modification of histone H2AX that is measured as γ-H2AX foci in the nucleus is an early event of DNA DSBs [41]. Pre-treatment of BEAS-2B cells with test compounds reduced the NNKAc-mediated histone variant reorganization which regulates DNA methylation [42]. This could have resulted in the observed similarities between reduced levels of NNKAc-induced γ-H2AX foci and DNA fragmentation levels with pre-treatment of test compounds [31]. DNA fragmentation is an irreversible phenomenon that leads to cell death [42]. The levels of DNA fragmentation seen in untreated BEAS-2B cells could be due to normal cellular homeostatic mechanisms to remove large DNA fragments from dying cells [43]. The alkaline comet assay is used to measure the effects of carcinogenic substances such as NNKAc on both DNA DSBs and SSBs [44]. NNKAc can damage all DNA bases (DNA base damage potential: guanine > adenine > cytosine > thymine) by forming bulky POB-DNA adducts and causing both SSBs and DSBs [30,45].

Luteolin, chrysin, cyanidin, and non-flavonoid compound curcumin in the current study were able to reduce the NNKAc-induced DNA damage in terms of both DNA DSBs and SSBs (alkaline comet assay) and DNA fragmentation levels at concentrations that reduced the NNKAc-induced ROS levels. However, for the significant reduction (*p* < 0.05) of NNKAc-induced γ-H2AX foci in BEAS-2B, comparatively higher concentrations of luteolin, chrysin, and curcumin were required. Furthermore, a significant reduction (*p* > 0.05) in NNKAc-induced γ-H2AX foci was not observed with cyanidin at the tested concentrations (*p* > 0.05). The observed differences between the comet and γ-H2AX immunofluorescence assays could be due to the effectiveness of chrysin, luteolin, cyanidin, and curcumin in reducing NNKAc-induced DNA SSBs at comparatively low concentrations. Since the alkaline comet assay quantifies both SSBs and DSBs, to study the effects of chrysin, luteolin, cyanidin, and curcumin, the neutral comet assay that is specific for the DSBs should be further studied [46]. Furthermore, naringenin, procyanidin B2, and non-flavonoids such as DMF, resveratrol, catechol, and sulforaphane demonstrated reduced DNA damage determined by γ-H2AX, comet, and DNA fragmentation ELISA assays at concentrations similar to the concentrations that reduced NNKAc-induced ROS levels significantly (*p* < 0.05). In comparison, both genistein and quercetin were able to reduce the NNKAc-induced DNA damage at concentrations less than the concentrations that reduced ROS levels in NNKAc-induced BEAS-2B cells significantly (*p* < 0.05).

The activation of the Nrf2/ARE pathway plays a significant role in the prevention of DNA damage and possible carcinogenesis by managing oxidative stress via the expression of antioxidant defense enzymes and phase 2 detoxifying enzymes [17,47]. In general, the Nrf2/ARE pathway is activated by oxidative stress [25,31]. However, the Nrf2 protein can be activated by both canonical and non-canonical mechanisms [16]. Akt is an upstream non-canonical activator that also plays an important role in cell survival, cell proliferation, cell growth, and cell metabolism [48]. Phosphorylated Akt (*p*-Akt) activates Nrf2 protein by phosphorylating Nrf2 (p-Nrf2) at Ser40 residue [49]. Phosphorylation of Ser473 residue is facilitated by either a mammalian target of rapamycin (mTOR) 2 or DNA-PKs whereas Thre308 is phosphorylated by upstream kinases such as PI3K [50,51]. From the studies on reductions in NNKAc-induced DNA damage in BEAS-2B cells, quercetin, genistein, and procyanidin B2 were effective in reducing NNKAc-induced ROS and DNA damage in lower concentrations compared to other tested compounds. Therefore, the effects of quercetin, genistein, and procyanidin B2 and selected positive controls (DMF and hydrogen peroxide) on Nrf2/ARE signaling were further investigated to determine their involvement in upstream kinase Akt phosphorylation, p-Nrf2 nuclear translocation, and antioxidant enzyme activities.

Among tested positive controls, DMF is known to activate Nrf2 protein through canonical mechanisms such as the modification of Keap 1 Cys residues [52]. Therefore, the DMF-mediated Nrf2 activation could be independent of Akt phosphorylation as observed in the current study. However, Nrf2 phosphorylation at Ser40 can also be mediated by PKC [53]. Moreover, the hydrogen peroxide-mediated phosphorylation of Nrf2 protein in the current study could be dependent on Akt phosphorylation. Zhuang, et al., 2019 have previously demonstrated the upregulation of p-Akt (Ser473), nuclear Nrf2 levels, and cellular HO-1 protein levels against hydrogen peroxide treatment in intestinal epithelial IEC-6 cells [54]. However, the Akt phosphorylation can be bi-phasic with time which depends on the type of cell and the activator as demonstrated previously [55]. Furthermore, increased levels of nuclear-translocated p-Nrf2 were observed with hydrogen peroxide and DMF. The increased levels of nuclear p-Nrf2 could be due to the Nrf2 phosphorylation followed by stabilization which suppresses the Nrf2 ubiquitination-mediated degradation [56]. Interestingly, exogenous hydrogen peroxide substantially upregulated the catalase and GPx activities except SOD activity in BEAS-2B cells. Antioxidant defense enzymes such as catalase and GPx are known to neutralize cellular hydrogen peroxide levels by converting them into water [57]. However, to maintain the catalytic activity of GPx, several cofactors (reduced glutathione, NAD(P)H), and glutathione reductase) are required [57,58]. The ability of hydrogen peroxide to upregulate antioxidant defense enzymes (i.e., catalase) has been previously demonstrated at comparatively low concentrations (<50 µM) [59,60]. DMF treatment maintained the SOD, catalase, and GPx activities in BEAS-2B cells at basal levels which indicates that DMF does not affect the redox balance under normal physiological conditions.

In this study, quercetin, genistein, and procyanidin B2 were the most effective flavonoids at physiologically relevant low concentrations (0.1–1 μM) in reducing NNKAc-induced DNA damage and therefore were further studied for their potential mechanisms of activation of Nrf2/ARE pathway. Therefore, the effects of quercetin, genistein, and procyanidin B2 on the phosphorylation of Akt at Ser473 were studied. BEAS-2B cells treated only with quercetin or procyanidin B2 at the tested highest concentration (25 µM) exhibited a significantly higher (*p* < 0.05) level of p-Akt/Akt ratio. Lee and colleagues (2011) demonstrated that quercetin decreased cell viability (even at concentrations less than 20 µM) and p-Akt levels in BEAS-2B cells via downregulation of Akt-mediated cell survival [61]. However, the findings of the study conducted by Lee and colleagues in 2011 [61] are contradictory to the current study since BEAS-2B cell viability was not affected at tested concentrations of quercetin in addition to the observed increase (*p* > 0.05) in p-Akt/Akt ratio at the tested highest concentration. Similarly, Merlin, et al., 2021 have demonstrated that BEAS-2B cells treated with quercetin up to 100 µM (>95% cell viability) do not affect the cell viability [30]. Furthermore, quercetin does not affect the human oral keratinocyte cell viability up to 100 µM and upregulates the PI3K/Akt pathway to attenuate the LPS-induced cell injury [62].

The ability of quercetin, genistein, and procyanidin B2 to activate the Nrf2/ARE pathway has been previously demonstrated in numerous experimental models [63,64,65,66,67]. In this study, both quercetin and genistein treatments alone did not increase the p-Nrf2/Nrf2 ratio in BEAS-2B cells significantly (*p* > 0.05). In contrast, procyanidin B2 treatment alone was effective in increasing the p-Nrf2/Nrf2 ratio. Similar to the current study, procyanidin B2 has previously demonstrated its ability to upregulate Nrf2 proteins through the upregulation of p-Akt levels in alleviating cypermethrin-induced oxidative stress in cerebral cortical neurons of C57BL/6 mice [66]. In our experimental model, BEAS-2B cells treated with quercetin were not effective in p-Nrf2 nuclear translocation and antioxidant defense enzyme activities. In comparison to quercetin, both genistein and procyanidin B2 were able to upregulate the Nrf2 nuclear translocation and catalase activity in BEAS-2B cells. Since genistein and procyanidin B2 facilitated the p-Nrf2 nuclear translocation, the mechanisms of these flavonoids activating Nrf2 should be further investigated. GSK3β is a serine/threonine kinase that phosphorylates Nrf2 at Ser335 and Ser338 (mouse sequence) in the Neh6 domain which facilitates Nrf2 nuclear export in addition to the Nrf2 degradation [56]. In contrast, AMPK facilitates Nrf2 nuclear translocation through phosphorylation of Nrf2 at Ser558 which improves the Nrf2 stability [68,69]. Genistein-mediated reduction of hydrogen peroxide-induced ROS levels in human visceral adipocytes has been associated with the activation of Akt and AMPK [70]. Therefore, the effects of genistein and procyanidin B2 on the inhibition of GSK3β and upregulation of AMPK should be further studied. As described previously, Nrf2 activation can be facilitated by PKC [53]. Li, et al., 2018 showed that a methyl derivative of genistein (7-*O*-methylbiochanin A) mediated Nrf2 activation, and upregulation of NQO-1 protein levels are associated with the activation of PI3K, MAPK, PKC, and PERK pathways [71]. PKC and AMPK could have a complementary effect on Nrf2 activation and nuclear translocation in vitro [68]. Since a substantial effect on p-Akt level in genistein-treated BEAS-2B cells was not observed, the effects of genistein on PKC should be further investigated in a time-dependent manner.

## 4. Materials and Methods

### 4.1. Antibodies, Kits, Chemicals, and Reagents

Anti-Nrf2 rabbit (Catalogue number [Cat]: ab62352) and anti-phospho-Nrf2 (Ser40) rabbit (Cat: ab76026) primary antibodies were purchased from Abcam Inc., (Toronto, ON, Canada). Anti-phospho-histone H2AX (ser139) mouse (Cat: 05-636) primary antibody was purchased from Sigma-Millipore (Etobicoke, ON, Canada). Anti-Akt rabbit (Cat: 92725), anti-phospho-Akt rabbit (Ser473) (Cat: 4060T) primary antibodies, horseradish peroxidase (HRP)-linked anti-rabbit secondary antibody (Cat: 7074P2) and HRP conjugated anti-β-actin rabbit (Cat: 12620S) antibody were purchased from Cell Signaling Technology, Inc., (Danvers, MA, USA). Alexa Fluor^®^ 594 donkey anti-mouse (Cat: A-21203) and Alexa FlourTM 488 goat anti-rabbit (Cat: A11034) secondary antibodies were purchased from Thermo Fisher Scientific (Chelmsford, MA, USA).

Comet SCGE assay kit (Cat: ADI-900-166) was purchased from Enzo (New York, NY, USA). Cellular DNA fragmentation ELISA kit ((Cat: 11585045001) was purchased from Roche Diagnostics, Mannheim, Berlin, Germany). Superoxide dismutase (SOD) activity (Cat: ab65354), catalase activity (Cat: ab83464), and glutathione peroxidase (GPx) activity (Cat: ab219926) assay kits were purchased from Abcam Inc. (Toronto, ON, Canada).

Ascorbic acid (Cat: A5960), beta carotene (Cat: 22040), caffeic acid (Cat: C0625), catechol (Cat: 135011), chlorogenic acid (Cat: C3878), chrysin (Cat: 95082), curcumin (Cat: C1386), cyanidin-3-*O*-glucoside (Cat: PHL89616), cyanidin chloride (Cat: 528-58-5), dimethyl fumarate (DMF) (Cat: 242926), epicatechin (Cat: E1753), genistein (Cat: 4478-93-7), isorhamnetin (Cat: 17794), luteolin (Cat: 491-70-3), methyl 4-hydroxybenzoate (Cat: H5501), naringenin (Cat: 52186), phloretin (Cat: 60-82-2), phloridzin dihydrate (Cat: P3449), phloroglucinaldehyde (Cat: T65404), protocatechuic acid (Cat: 08992), quercetin (Cat: Q4951-109), quercetin-3-*O*-glucuronic acid (Cat: 22688-79-5), resveratrol (Cat: R5010), and sulforaphane (Cat: 4478-93-7), 2′ 7′-Dichlorofluorescein diacetate (DCFDA) (Cat: D6883), and dimethyl sulfoxide (DMSO) (Cat: 276855) were purchased from Sigma-Aldrich (Oakville, ON, Canada). Procyanidin B2 (Cat: 29106-49-8) was purchased from Chengdu Alfa Biotechnology Co., Ltd. (Pixian, Chengdu, China). 4-[(Acetoxymethyl) nitrosamino]-1-(3-pyridyl)-1-butanone (NNKAc) (Cat: 167550) was purchased from Toronto Research Chemicals (Toronto, ON, Canada). LHC-8 growth medium (Cat: 12678017) was purchased from Thermo Fisher Scientific (Chelmsford, MA, USA). Cell Titer 96^®^ AQueous MTS reagent powder (Cat: G1111) was purchased from Promega (Madison, WI, USA). Vectashield^®^ containing 4ʹ,6-diamidino-2-phenylindol (DAPI) (Cat: H-1200) was purchased from Vector Laboratories Inc. (Burlingame, CA, USA). All the chemicals used in this study were of analytical grade and suitable for cell-based experiments.

### 4.2. Cell Culture

The normal bronchial epithelial cell line, BEAS-2B (ATCC^®^ CRL-9609TM) was purchased from the American Tissue Type Culture Collection (Manassas, VA, USA). The BEAS-2B cells were cultured in the LHC-8 medium supplemented with 5% FBS, 100 U/mL penicillin, and 100 μg/mL streptomycin in T-75 polystyrene culture flasks (75 cm^2^) at 37 °C and 5% CO_2_ in an incubator maintained at 100% humidity. Prior to use, culture flasks were coated with a mixture of fibronectin (0.01 mg/mL), bovine serum albumin (0.01 mg/mL) and bovine collagen type 1 (0.03 mg/mL) in phosphate-buffered saline (PBS) overnight. Cells grown up to 80% confluence and cell passages between 5 and 25 were used in the experiments.

### 4.3. Measurement of Intracellular ROS

The ROS levels in the BEAS-2B cells were determined following treatments according to the method described by Wang and Joseph (1999) [72]. Cells were seeded in black 96-well microplates at a density of 1 × 10^4^ per well and incubated for 24 h. Cells were pre-treated with six concentrations (0.1, 1, 5, 10, 25, and 50 µM) of selected compounds for 3 h. Pre-treated cells were exposed to 100 µM NNKAc for another 3 h to induce ROS generation. DMSO (0.1% or 0.4%) was used as the vehicle control. Following treatments, 5 μM 2′ 7′-dichlorofluorescein diacetate (DCFDA) was added to each well and incubated for 30 min at dark. The fluorescence intensity was measured at an excitation wavelength of 485 nm and an emission wavelength of 535 nm using a microplate reader (Infinite^®^ 200 PRO, Tecan Trading AG, Mannedorf, Switzerland).

### 4.4. Cell Viability by Cell Titer 96™ Cell Viability Assay

The cell viability of BEAS-2B was determined using the Cell Titer 96™ cell viability assay (MTS), as described by Wang et al., 2010. [73]. Cells were seeded in a clear flat-bottom 96-well microplate at a density of 1 × 10^4^ cells per well and incubated for 24 h overnight. After 24 h, cells were pre-treated with 5 concentrations (0.1, 1, 5, 10, and 25 µM) of selected compounds for 3 h. To induce ROS generation, pre-treated cells were exposed to 100 µM NNKAc for another 3 h. DMSO (0.1% or 0.4%) was used as the vehicle control. Blanks for the experiment were conducted without cells but with tested treatment. Following treatments, the MTS reagent with PMS (20:1) was added to each well (15 µL/well) and incubated for 3 h in the dark. Absorbance was measured at 490 nm using a microplate reader (Infinite^®^ 200 PRO, Tecan Trading AG, Mannedorf, Switzerland). Results were expressed in terms of percentage cell viability using the following formula, A is the absorbance of the treated cells, B is the absorbance of medium control with cells, C is the absorbance of the blank of treated compounds, and D is the absorbance of medium without cells.


$$\mathrm{Percentage}\;\mathrm{cell}\;\mathrm{viability}=\left(\frac{A-C}{B-D}\right)\times 100$$

### 4.5. γ-H2AX Immunofluorescence Assay

DNA damage at histone levels was measured using the immunofluorescence assay previously described by Ivashkevich et al., 2012 [42] by quantifying γ-H2AX foci in BEAS-2B cells. Initially, 1 × 10^5^ cells per well were seeded on a sterilized coated coverslip placed in a clear flat-bottom 6-well plate followed by a 24-h incubation. After 24 h, cells were treated with selected test compounds and NNKAc as explained before (Section 4.4). Following treatments, cells were washed with PBS (×1) and fixed with 3.7% paraformaldehyde for 20 min in the dark. Then, cells were permeabilized with 0.5% Triton X-100 in PBS (×1) for another 15 min at room temperature. Then, coverslips were blocked using 4% BSA for 20 min at room temperature. After blocking, coverslips were incubated with anti-phospho-histone H2AX primary antibody (1:250 ratio) for 1 h at room temperature. Subsequently, coverslips were washed three times with PBS and incubated with the secondary antibody (Alexa fluorophore^®^ 594 donkey anti-mouse) (1:500 ratio) for another 45 min at room temperature in the dark. Once excessive secondary antibodies on coverslips were washed three times with PBS (×1), wet mounting of coverslips onto glass slides was performed using Vectashield^®^ containing 4′,6-diamidino-2-phenylindol (DAPI), the wet-mounting medium. Coverslips were sealed with clear nail polish and dried in the dark at room temperature. Images of slides were taken using a fluorescence microscope (EVOSTM FLoid Imaging System, Bothell, WA, USA) at 100× magnification. The number of phosphorylated histone-H2AX foci was quantified for at least 50 nuclei per treatment using ImageJ software (Version 1.53k, National Institute of Mental Health, Bethesda, MD, USA).

### 4.6. Comet Assay

The DNA damage in BEAS-2B cells was determined by single-cell gel electrophoresis assay using the Comet SCGE assay kit (Cat: ADI-900-166, Enzo, New York, NY, USA). Initially, 1 × 10^5^ cells per well were seeded in a clear flat-bottom 6-well plate followed by a 24 h incubation. After 24 h, cells were treated with selected compounds and NNKAc as explained before (Section 4.4). Following treatments, harvested cells were mixed with molten low-melting agarose at a ratio of 10: 1 by volume. Then, 30 µM of each sample was inserted into a well in a 20-well comet slide (Cat: 4252-500-01, R & D Systems, Minneapolis, MN, USA) immediately and was kept at 4 °C under dark conditions for 20 min to solidify. The cold lysis buffer was used to immerse the slides for 45 min at 4 °C. The slides were then subjected to an alkaline treatment (pH > 13) consisting of 300 mM NaOH and 1 mM EDTA and incubated for 45 min in the dark at room temperature. The slides were washed once with 1× TBE buffer for 5 min and subjected to horizontal electrophoresis conditions of 1 V/cm for 11 min in 1× TBE buffer. Following electrophoresis, slides were immersed in ethanol (70%) for 5 min and air-dried. Slides were stained with CYGREEN^®^ dye in a ratio of 1:1000 and imaged using fluorescence microscopy (EVOSTM FLoid Imaging System; Bothell, WA, USA) at 100× magnification. The OpenComet plugin in ImageJ software (Version 1.53k, National Institutes of Mental Health, Bethesda, MD, USA) was used to calculate the tail moment of the DNA. A minimum of 30 cells were quantified for each treatment.

### 4.7. DNA Fragmentation Analysis

Cellular DNA fragmentation ELISA kit (Ref # 11585045001, Roche Diagnostics, Mannheim, Berlin, Germany) [74] was used to determine the DNA fragmentation in BEAS-2B cells. Initially, 1 × 10^5^ cells/mL were labeled with bromodeoxyuridine (BrdU) (10 μΜ) in the culture medium for 2 h. A hundred microliters of BrdU labelled cells were seeded in clear flat 96-well plates and incubated for 24 h. After 24 h, cells were treated as mentioned before (Section 4.4). Treated cells were lysed with lysis solution prior to centrifugation at 1000× *g* for 5 min. Then, 100 μL of supernatants from each treatment were transferred to anti-DNA coated microplates and incubated overnight at 4 °C. Next, the microplate was subjected to microwave irradiation at 500 W for 5 min to denature DNA. Once the microplate was cooled down to room temperature, the anti-BrdU-peroxidase (POD) conjugate solution (100 μL/well) was added and incubated for 90 min at room temperature. Then, the microplate was washed thrice using the washing buffer followed by the addition of 100 μL substrate solution. The microplate was incubated for 5 min in the dark on a shaker for color development, and the stop solution (concentrated sulfuric acid) (25 μL/well) was added. After that, the microplate was incubated for another minute on the shaker, and the absorbance was recorded using a microplate reader (Infinite^®^ 200 PRO, Tecan Trading AG, Mannedorf, Switzerland) at a wavelength of 450 nm.

### 4.8. Western Blot Analysis

Western blot analysis was performed to study the effect of quercetin, genistein, and procyanidin B2 on the phosphorylation of Akt and Nrf2 as described by George and Rupasinghe, 2017 [31]. Initially, BEAS-2B cells were treated with two concentrations (1 and 25 μM) of quercetin, genistein, and procyanidin B2 for 3 h. The cells treated with 25 μM DMF, and 10 μM hydrogen peroxide for 3 h were used as positive controls, and 0.1% DMSO was used as the vehicle control. Following the treatments, cells were harvested. Then for cell lysis, a mixture of radio-immunoprecipitation assay (RIPA) buffer (0.1% sodium dodecyl sulfate [SDS], 5 mM EDTA, 25 mM Tris-HCl [pH-7.6], 1% Triton X-100, 1 % sodium deoxycholate, and 150 mM NaCl) and protease inhibitor cocktail mixed at a ratio of 10:1 was added to the cell pellet and incubated for 30 min on ice. Following cell lysis, Pierce™ Coomassie (Bradford) protein assay kit (Cat: 23200, Thermo Fisher Scientific, Rockford, IL, USA) was used to estimate the protein content in extracted protein samples. Twenty micrograms of protein samples were loaded to 10% SDS-PAGE gel and gel electrophoresis was performed. Proteins separated in SDS-PAGE gels were electro-transferred onto a polyvinylidene difluoride (PVDF) membrane (Cat: 88518, Thermo Fisher Scientific, Rockford, IL, USA). Electro-transferred PVDF membranes were then blocked with commercially available 5% non-fat milk for 1 h at room temperature. Then, the blocked PVDF membrane was probed overnight with gentle shaking at 4 °C with specific primary antibodies (anti-phospho-Akt (Ser473), anti-Akt, and anti-Nrf2 rabbit primary antibodies at a ratio of 1:1000, and anti-phospo-Nrf2 (Ser40) rabbit antibody at a ratio of 1:5000). Following probing for primary antibodies, membranes were washed and re-probed with the HRP-linked anti-rabbit secondary antibodies (1:2000) for 1 h with gentle shaking on a rocker. For β-actin, HRP conjugated anti-β-actin rabbit antibody was probed overnight. Clarity™ and Clarity Max™ Western ECL Substrates Kit (Cat: 1705060, Bio-Rad Laboratories Inc., Hercules, CA, USA) were used to develop the membranes for imaging. The membranes probed to study p-Akt and p-Nrf2 were used to probe their respective total protein antibodies after stripping off the antibodies from the membrane. The protein expression of each band was normalized to their respective β-actin band intensity. Results were expressed as the phosphorylated protein expression: total protein expression relative to the control.

### 4.9. p-Nrf2 Nuclear Translocation by Immunofluorescence Assay

The effect of quercetin, genistein, and procyanidin B2 on p-Nrf2 nuclear translocation was evaluated using an immunofluorescence assay. The immunofluorescence assay protocol identified in Section 4.5 was followed to study the level of p-Nrf2 nuclear translocation with minor modifications. Cells were treated as mentioned in the Section 4.8. Anti-phospho-Nrf2 (Ser40) was used as the primary antibody at a ratio of 1:200 in 4% BSA, and Alexa FlourTM 488 goat anti-rabbit secondary antibody was used at a ratio of 1:500 in 4% BSA. Images of slides were taken using a fluorescence microscope (EVOSTM FLoid Imaging System, Bothell, WA, USA) at 100× magnification. The corrected total nuclear fluorescence (CTNF) values of at least 30 nuclei per treatment were measured using ImageJ software (Version 1.53k, National Institute of Mental Health, Bethesda, MD, USA). CTNF values were calculated using the following formula, A is the area of the selected nucleus whereas MC is the mean fluorescence value of the cell, and MB is the mean fluorescence value of the background.


$$\mathrm{Corrected}\;\mathrm{total}\;\mathrm{nuclear}\;\mathrm{fluorescence}\;\mathrm{value}\;\left(\mathrm{CTNF}\right)=\left(A\times \mathrm{MC}\right)-\left(A\times \mathrm{MB}\right)$$

### 4.10. Superoxide Dismutase Activity

A superoxide dismutase (SOD) activity assay kit (Cat: ab65354, Abcam Inc, Toronto, ON, Canada) was used to determine the SOD activity in BEAS-2B cells as per the manufacturer’s instructions. Cells were treated as mentioned in Section 4.8. After treatments, cells were harvested and were lysed using ice-cold 0.1 M Tris-HCl lysis buffer (pH 7.4, 0.5% Triton X-100, 5 mM β-mercaptoethanol, and 0.1 mg/mL phenylmethylsulfonyl fluoride protease inhibitor). A clear flat-bottom 96-well plate was used to perform the assay. Reaction wells consisted of three types of blanks in addition to the sample wells. Blank 1 and blank 3 were loaded with 20 µL of deionized water, whereas both blank 2 and sample wells were loaded with 20 µL of extracted proteins in lysis buffer. Then, 200 µL of WST solution provided with the kit was added to each well, followed by the addition of 20 µL of dilution buffer to blanks 2 and 3. Twenty microliters of SOD enzyme solution were then added to sample wells and blank 1 well. The reaction mixtures were incubated for 20 min at 37 °C. The absorbance was recorded at 450 nm using a microplate reader Infinite^®^ 200 PRO, Tecan Trading AG, Mannedorf, Switzerland). SOD activity in terms of percentage inhibition rate was calculated using the following formulae, whereas Ab1, Ab2, Ab3, and As are the absorbance values of blank 1, blank 2, blank 3, and samples, respectively.


$$\mathrm{SOD}\;\mathrm{activity}\;\left(\%\;\mathrm{inhibition}\;\mathrm{rate}\right)=\left(\frac{\left(\mathrm{Ab}1-\mathrm{Ab}3\right)-\left(\mathrm{As}-\mathrm{Ab}2\right)}{\left(\mathrm{Ab}1-\mathrm{Ab}3\right)}\right)\times 100$$

### 4.11. Catalase Activity

The catalase activity assay kit (Cat: ab83464, Abcam Inc, Toronto, ON, Canada) was used to determine the catalase activity in BEAS-2B cells as per the manufacturer’s instructions. Cells were treated as mentioned in Section 4.8. After treatments, cells were harvested and lysed using the ice-cold catalase assay buffer provided with the kit. Hydrogen peroxide was used as the standard compound to determine the catalase activity. A stock solution of 20 mM hydrogen peroxide was used to prepare solutions of 0, 2, 4, 6, 8, and 10 nmol/well to generate the standard solution series. A clear flat-bottom 96-well plate was used to load the samples, whereas 90 µL/well of standard solutions and 5 µL/well of protein extracts were loaded to sample wells (catalases active sample wells) and sample high control wells (samples that were inhibited for catalase activity). The volumes of sample wells and sample high control wells were then adjusted to 78 µL/well with catalase assay buffer. The stop solution (10 µL/well) was added to both hydrogen peroxide standard wells and sample high control wells and incubated at room temperature for 5 min to inhibit the catalase activity. Fresh 1 mM hydrogen peroxide (12 µL/well) was added to sample wells and sample high control wells and incubated for 30 min at room temperature. After incubation, 10 µL/well of stop solution was added to each sample well. Then, a volume of 50 µL/well of the developer mix containing HRP solution, OxiRed probe, and catalase assay buffer at a ratio of 1:1:23 was added to each well and incubated for 10 min at room temperature. The absorbance was recorded at 570 nm using a microplate reader (Infinite^®^ 200 PRO, Tecan Trading AG, Mannedorf, Switzerland). The standard curve was used to calculate the reacted hydrogen peroxide amount in the sample using the absorbance values obtained by subtracting the absorbance of sample wells from the sample high control wells. One unit of catalase activity represents the amount of catalases that decomposes 1 µM of hydrogen peroxide per min at room temperature at pH 4.5. The catalase activity (mU/mL) of samples was calculated using the following formula, whereas B is the amount of hydrogen peroxide in the sample well calculated from the standard curve (nmol), 30 is the catalase reaction time (min), V is the sample volume added into the reaction volume (mL), and D is the dilution factor.


$$\mathrm{Catalase}\;\mathrm{activity}=\left(\frac{B}{30\times V}\right)\times D$$

### 4.12. Glutathione Peroxidase Activity

Glutathione peroxidase (GPx) activity assay kit (Cat: ab219926, Abcam Inc, Toronto, ON, Canada) was used to determine the GPx activity in BEAS-2B cells as per the manufacturer’s instructions. Cells were treated as mentioned in the Section 4.8. After treatments, cells were harvested and were lysed using ice-cold 0.1 M Tris-HCl lysis buffer (pH 7.4, 0.5% Triton X-100, 5 mM β-mercaptoethanol, and 0.1 mg/mL phenylmethylsulfonyl fluoride protease inhibitor). GPx enzyme was used as the standard compound to determine the GPx activity. A stock solution of 10 U/mL GPx was used to prepare solutions of 0, 0.625, 1.25, 2.5, 5, 10, 20, and 40 mU/mL to generate the standard solution series. A black clear flat-bottom 96-well plate was used to perform the assay, and 50 µL of PBS and 50 µL standard solutions were added to blank control wells and standard wells, respectively. The sample wells were loaded with 2 µL of extracted protein samples and the volumes of sample wells were adjusted with PBS up to 50 µL. Then, 50 µL of GPx assay mixture containing 100× GSH stock solution and enzyme mix solution at a ratio of 1:1 was added to each well. The reaction mixture was then incubated for 30 min in the dark at room temperature. After incubation, 20 µL of NADP sensor probe in PBS (1:20 ratio) and 20 µL of NADP assay solution were added to each well. The reaction mixture was incubated for 10 min in the dark at room temperature. After 10 min, 15 µL of enhancer solution was added to the reaction volume, and fluorescence increase was measured at an excitation wavelength of 420 nm and an emission wavelength of 480 nm using a microplate reader (Infinite^®^ 200 PRO, Tecan Trading AG, Mannedorf, Switzerland) in kinetic mode for every 2 min, for at least 30 min. The reaction rate (ΔRFU) was calculated as follows, whereas T1 and T2 are the chosen time points in minutes in the linear phase of the reaction progress curve. RFU1 and RFU2 values are the fluorescence values of the sample at T1 and T2 time points, and RFUb1 and RFUb2 are the fluorescence values of blanks at T1 and T2 time points.


$$\Delta \mathrm{RFU}=\left[\frac{\left(\mathrm{RFU}2-\mathrm{RFUb}2\;\right)–\left(\mathrm{RFU}1-\mathrm{RFUb}1\right)}{\left(T2-T1\right)}\right]$$

GPx activity (mU/mL) of the original samples was then calculated using the following equation, where B is the GPx activity in sample wells calculated using ΔRFU and GPx standard curve, Vw is the total volume of the well after reaction, Vs is the original sample volume added to the reaction mixture, and D is the dilution factor.


$$\mathrm{GPx}\;\mathrm{activity}=B\times \left(\frac{\mathrm{Vw}}{\mathrm{Vs}}\right)\times D$$

### 4.13. Experimental Design and Statistical Analysis

A complete randomized design was used for all experiments. Results were presented as mean values with standard deviation (±SD) relative to medium control. Analysis of variance was performed using one-way analysis of variance (ANOVA), and mean comparison was performed using Tukey’s multiple mean comparisons at *p* < 0.05 using Minitab 19 statistical software (LLC, Pennsylvania, USA). Screening of (poly)phenols for the effects on NNKAc-induced ROS was performed in triplicates and independently, two times. MTS assay, DNA fragmentation ELISA, catalase activity, GPx activity, and SOD activity assays were performed in duplicates and independently three times. All other experiments were performed in triplicates and independently, at least three times.

## 5. Conclusions

The investigated flavonoids (genistein, quercetin, luteolin, chrysin, cyanidin, naringenin, and procyanidin B2), flavonoid metabolites (isorhamnetin), simple (poly)phenols (catechol), stilbenes (resveratrol), curcuminoids (curcumin), and non-phenolics (sulforaphane and DMF) protected normal lung epithelial BEAS-2B cells from in vitro carcinogen insult. Evaluation of the most effective flavonoids (quercetin, genistein, and procyanidin B2) on the mechanisms involved in protecting BEAS-2B cells revealed that genistein and procyanidin B2 but not quercetin activated the Nrf2/ARE pathway. The procyanidin B2 mediated activation of the Nrf2/ARE pathway in terms of Nrf2 phosphorylation could be associated with the phosphorylation of Akt. Both genistein and procyanidin B2 upregulated p-Nrf2 nuclear translocation and catalase activity in BEAS-2B cells. Therefore, exerted protective effects of genistein and procyanidin B2 against carcinogen-induced ROS and DNA damage could be due to the activation of the Nrf2/ARE pathway by genistein and procyanidin B2. Moreover, consumption of diets rich in quercetin (i.e., onions, apples, and parsley), genistein (i.e., soy-based food), and procyanidin B2 (i.e., cocoa-based food, grape seeds, plums, and berries) could provide a protective effect against carcinogen-induced cancer. Since the protective effects of genistein, quercetin, and procyanidin B2 in BEAS-2B cells were also observed at physiologically relevant concentrations, a flavonoid-inspired functional food and/or a nutraceutical using the effective physiologically relevant concentrations of quercetin, genistein, and procyanidin B2 can be developed and assessed to reduce the risk of cancer. Additionally, the ability of dietary flavonoids to activate the Nrf2/ARE pathway in exerting protective effects against other chronic disorders such as neurodegenerative diseases and diabetes mellitus that are associated with oxidative stress should be further studied.

## Data Availability

All data presented in this study have been provided as Figures and Supplementary Materials.

## Supplementary Materials

The following supporting information can be downloaded at: https://www.mdpi.com/article/10.3390/ijms24043676/s1.
