# Supplementary material for: Genistein and Procyanidin B2 Reduce Carcinogen-Induced Reactive Oxygen Species and DNA Damage through the Activation of Nrf2/ARE Cell Signaling in Bronchial Epithelial Cells In Vitro
Source: Int J Mol Sci. 2023 Feb 12;24(4):3676. doi: 10.3390/ijms24043676 (PMC9961944; doi:10.3390/ijms24043676)
Supplement: Supplementary file 1 [file ijms-24-03676-s001.zip › ijms-2210899-supplementary.pdf]

International Journal of
Molecular Sciences

Supplementary Data

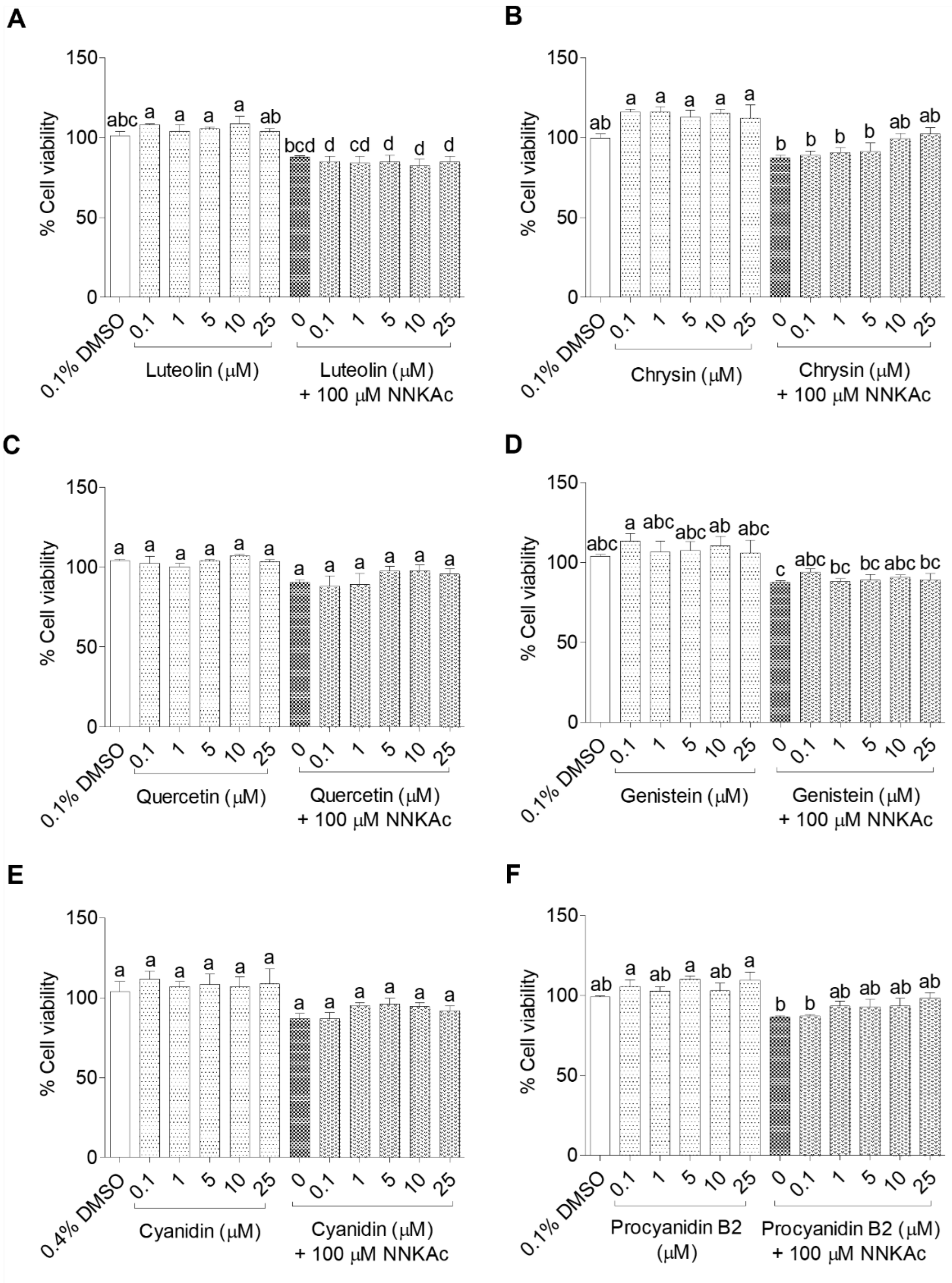

Figure S1. Effect of luteolin (A), chrysin (B), quercetin (C), genistein (D), cyanidin (E), and procyanidin B2 (F) on cell viability against NNKAc in BEAS-2B cells.

Cells were pre-treated with concentrations ranging from 0.1- 25 μM of selected compounds for 3 h. Pre-treated cells were exposed to 100 μM NNKAc for another 3 h. DMSO (0.1% or 0.4%) was used as the vehicle control. Effects on cell viability were quantified using the MTS assay. Three independent studies were performed, and results were expressed as mean ± standard deviation. Statistical analysis of data was performed by one-way ANOVA and mean comparison was done by Tukey's mean comparison method (α=0.05) using Minitab 19 statistical software (LLC,

Pennsylvania, USA). Mean values that do not share similar letters in bar graphs are significantly different (p<0.05). Abbreviations: NNKAc: 4-[(acetoxymethyl)nitrosamino]-1-(3-pyridyl)-1-butanone, DMSO: dimethyl sulfoxide.

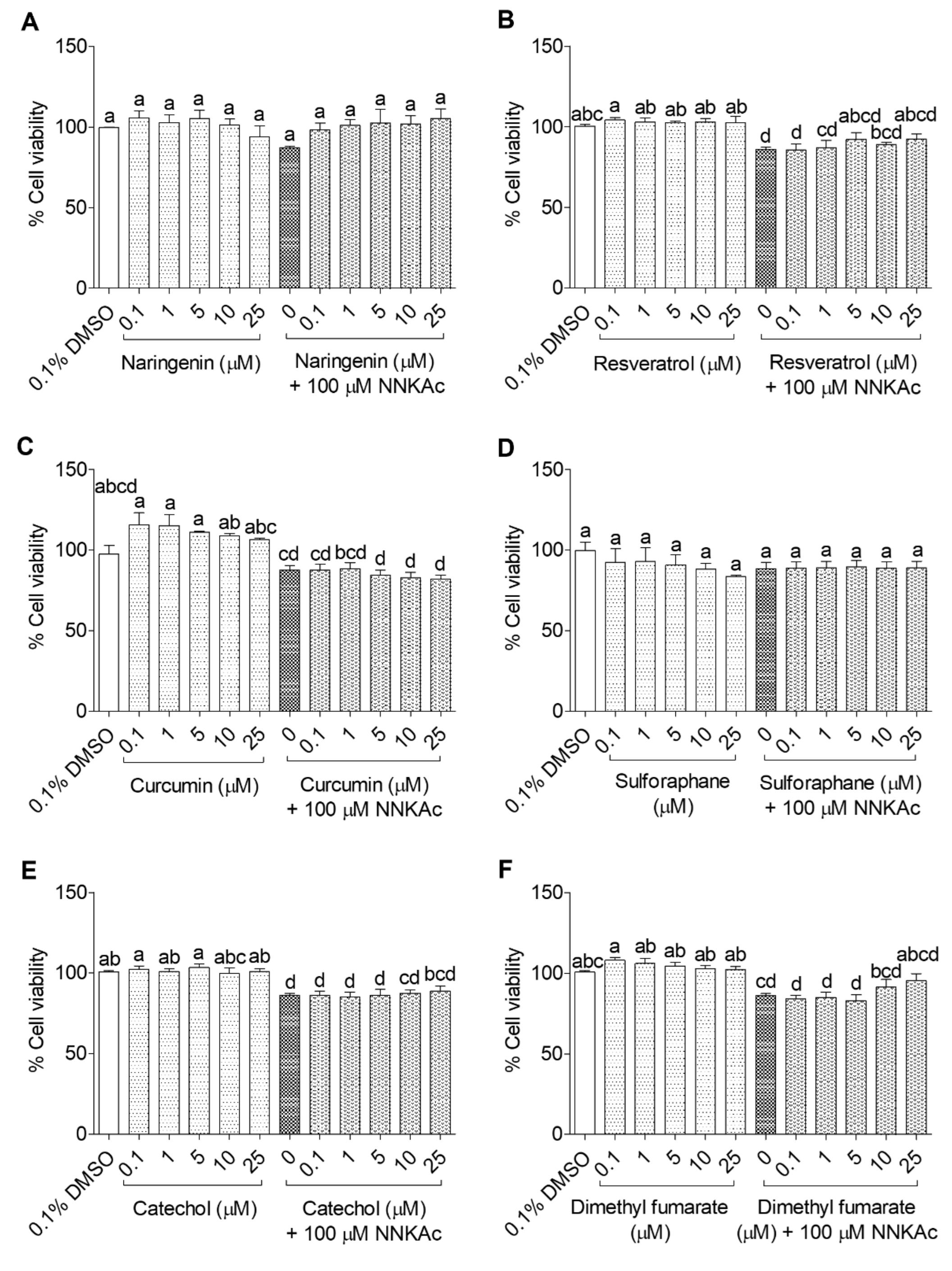

Figure S2: Effect of naringenin (A), resveratrol (B), curcumin (C), sulforaphane (D), catechol (E), and dimethyl fumarate (F) on cell viability against NNKAc in BEAS-2B cells.

Cells were pre-treated with concentrations ranging from 0.1- 25 μM of selected compounds for 3 h. Pre-treated cells were exposed to 100 μM NNKAc for another 3 h. DMSO (0.1%) was used as the vehicle control. Effects on cell viability were quantified using the MTS assay. Three independent studies were performed, and results were expressed as mean ± standard deviation. Statistical analysis of data was performed by one-way ANOVA and mean comparison was done by Tukey's mean comparison method (α=0.05) using Minitab 19 statistical software (LLC,

Pennsylvania, USA). Mean values that do not share similar letters in bar graphs are significantly different ($p<0.05$). Abbreviations: NNKAc: 4-[(acetoxymethyl)nitrosamino]-1-(3-pyridyl)-1-butanone, DMSO: dimethyl sulfoxide.

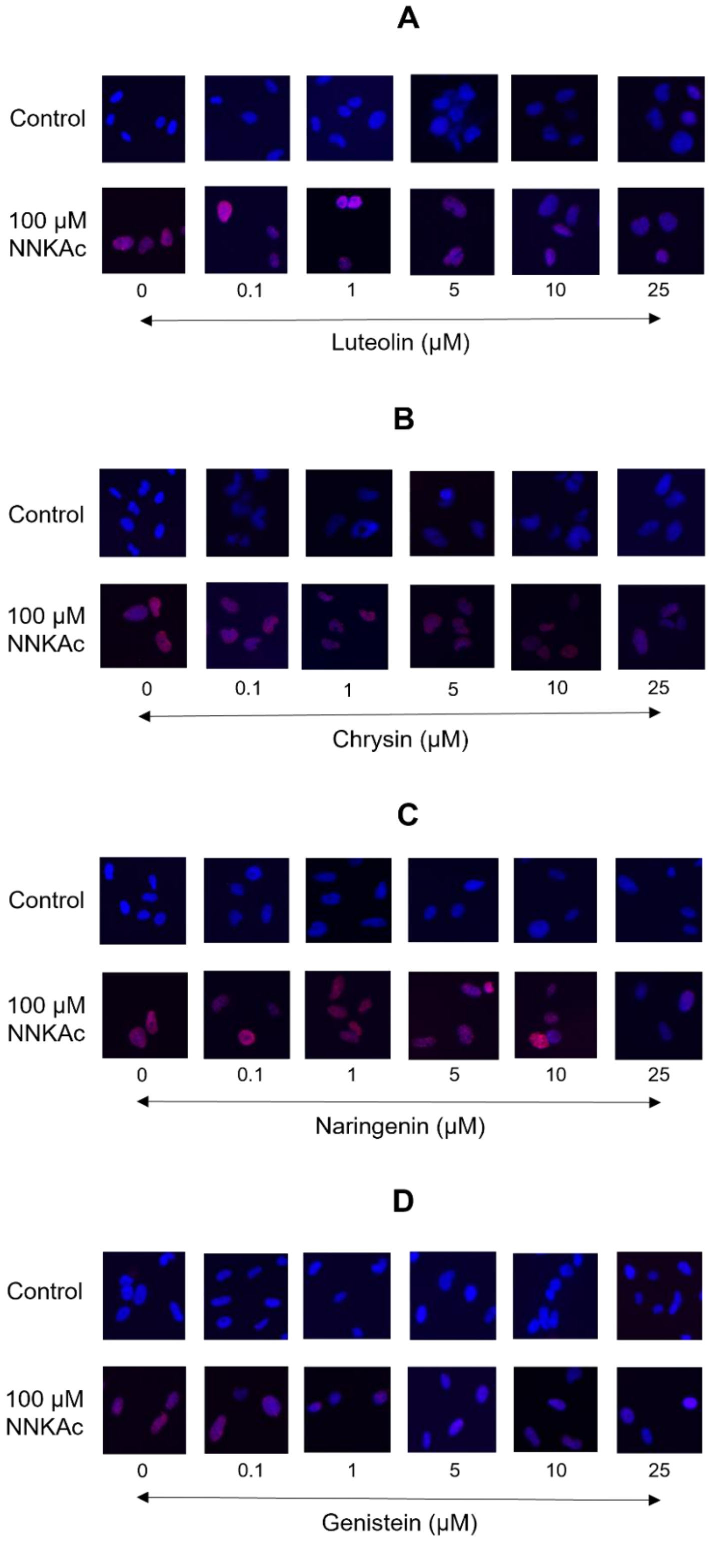

Figure S3: Effect of luteolin (A), chrysin (B), naringenin (C), and genistein (D) on NNKAc-induced DNA damage in BEAS-2B cells measured by γ-H2AX immunofluorescence assay. Abbreviations: NNKAc: 4-[(acetoxymethyl)nitrosamino]-1-(3-pyridyl)-1-butanone.

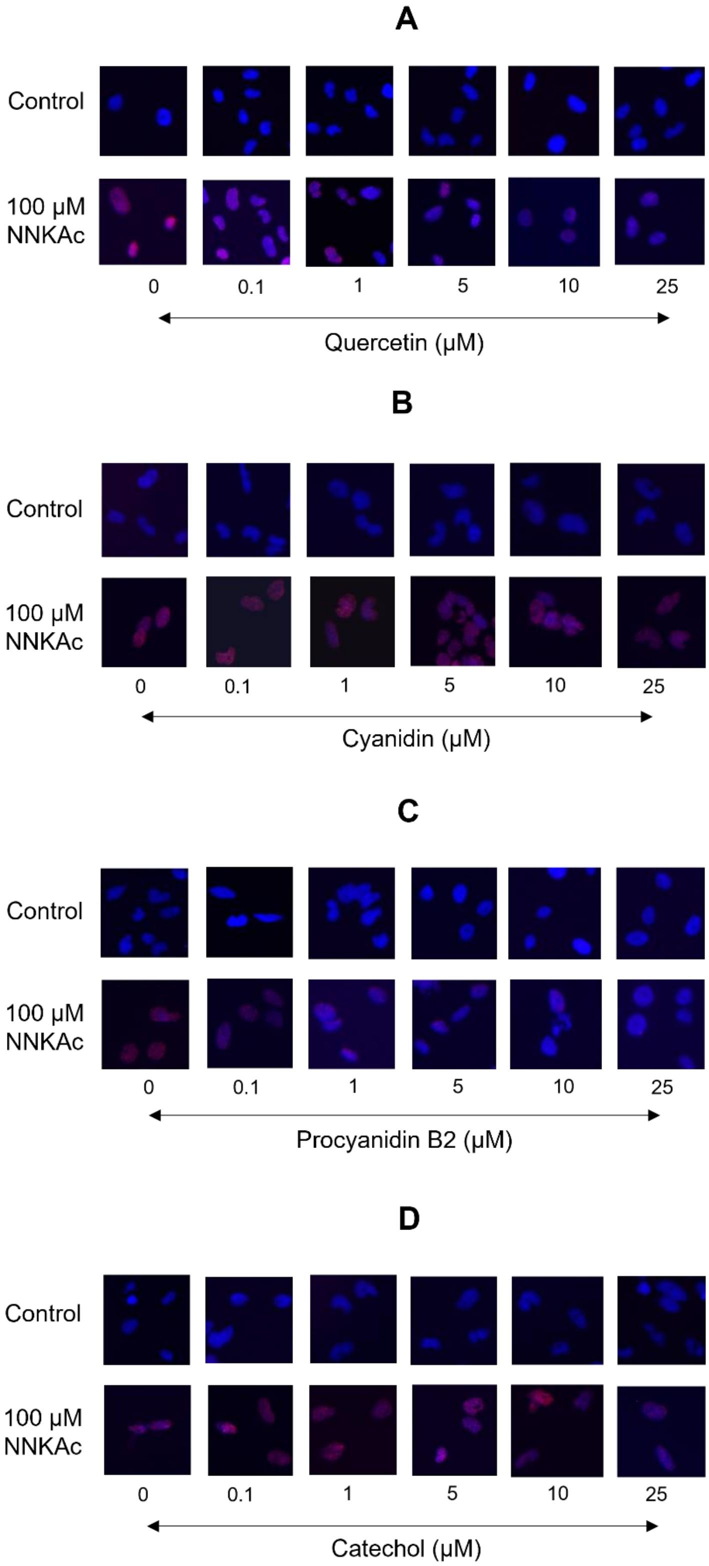

Figure S4: Effect of quercetin (A), cyanidin (B), procyanidin B2 (C), and catechin (D) on NNKAc-induced DNA damage in BEAS-2B cells measured by γ-H2AX immunofluorescence assay. Abbreviations: NNKAc: 4-[(acetoxymethyl)nitrosamino]-1-(3-pyridyl)-1-butanone.

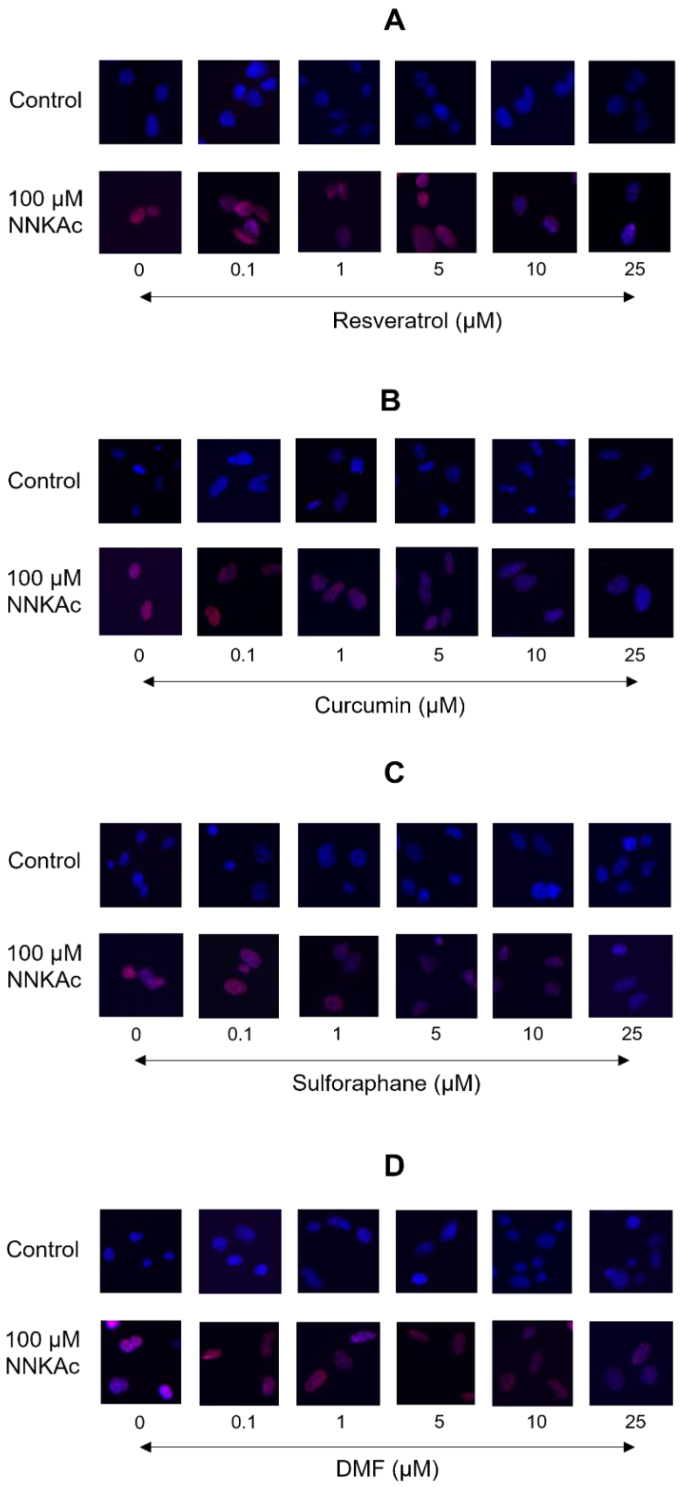

Figure S5: Effect of resveratrol (A), curcumin (B), sulforaphane (C), and dimethyl fumarate (D) on NNKAc-induced DNA damage in BEAS-2B cells measured by γ-H2AX immunofluorescence assay. Abbreviations: NNKAc: 4-[(acetoxymethyl)nitrosamino]-1-(3-pyridyl)-1-butanone and DMF: dimethyl fumarate.

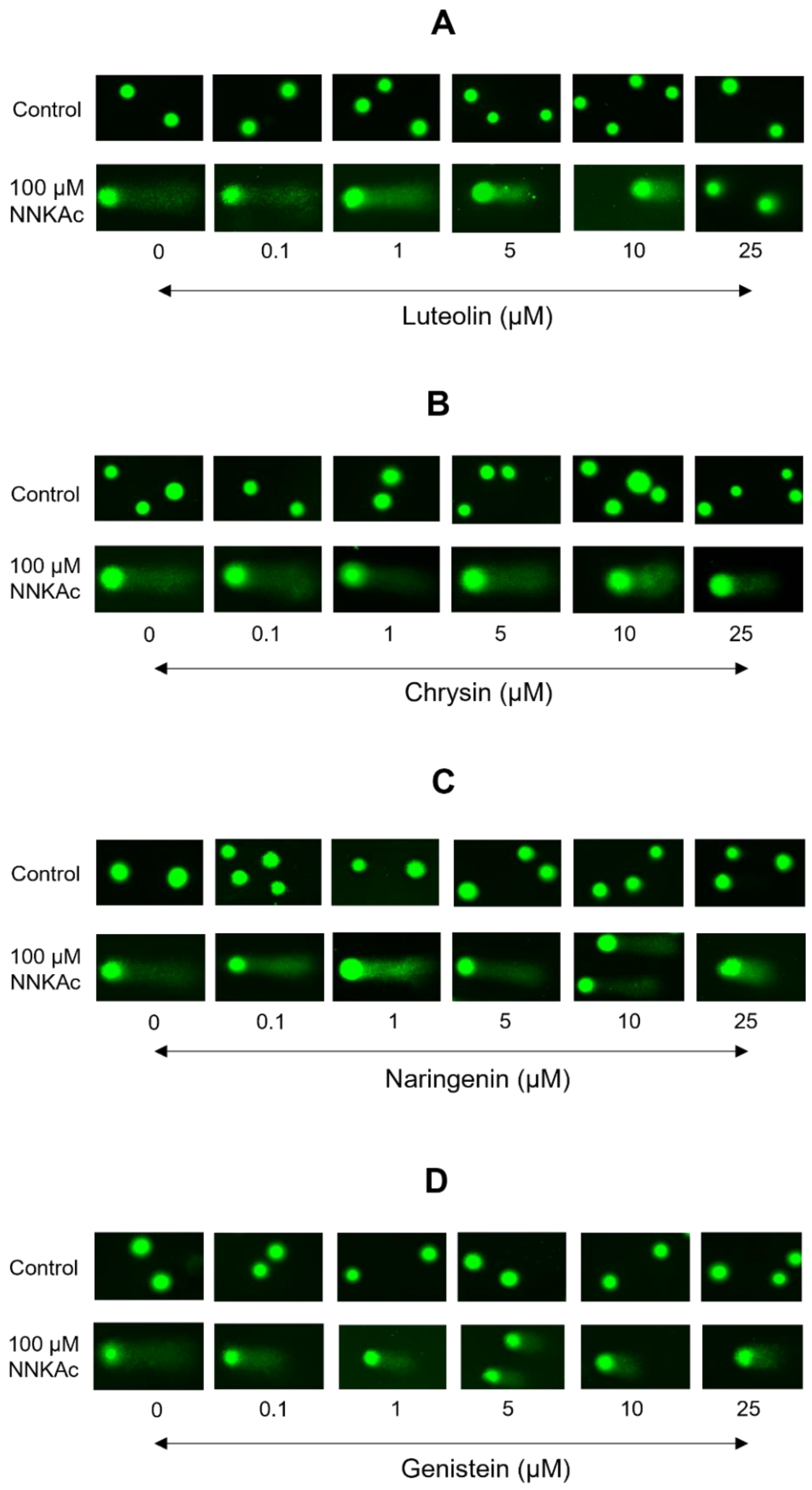

Figure S6: Effect of luteolin (A), chrysin (B), naringenin (C), and genistein (D) on NNKAc-induced DNA damage in BEAS-2B cells measured by comet assay. Abbreviations: NNKAc: 4-[(acetoxymethyl)nitrosamino]-1-(3-pyridyl)-1-butanone.

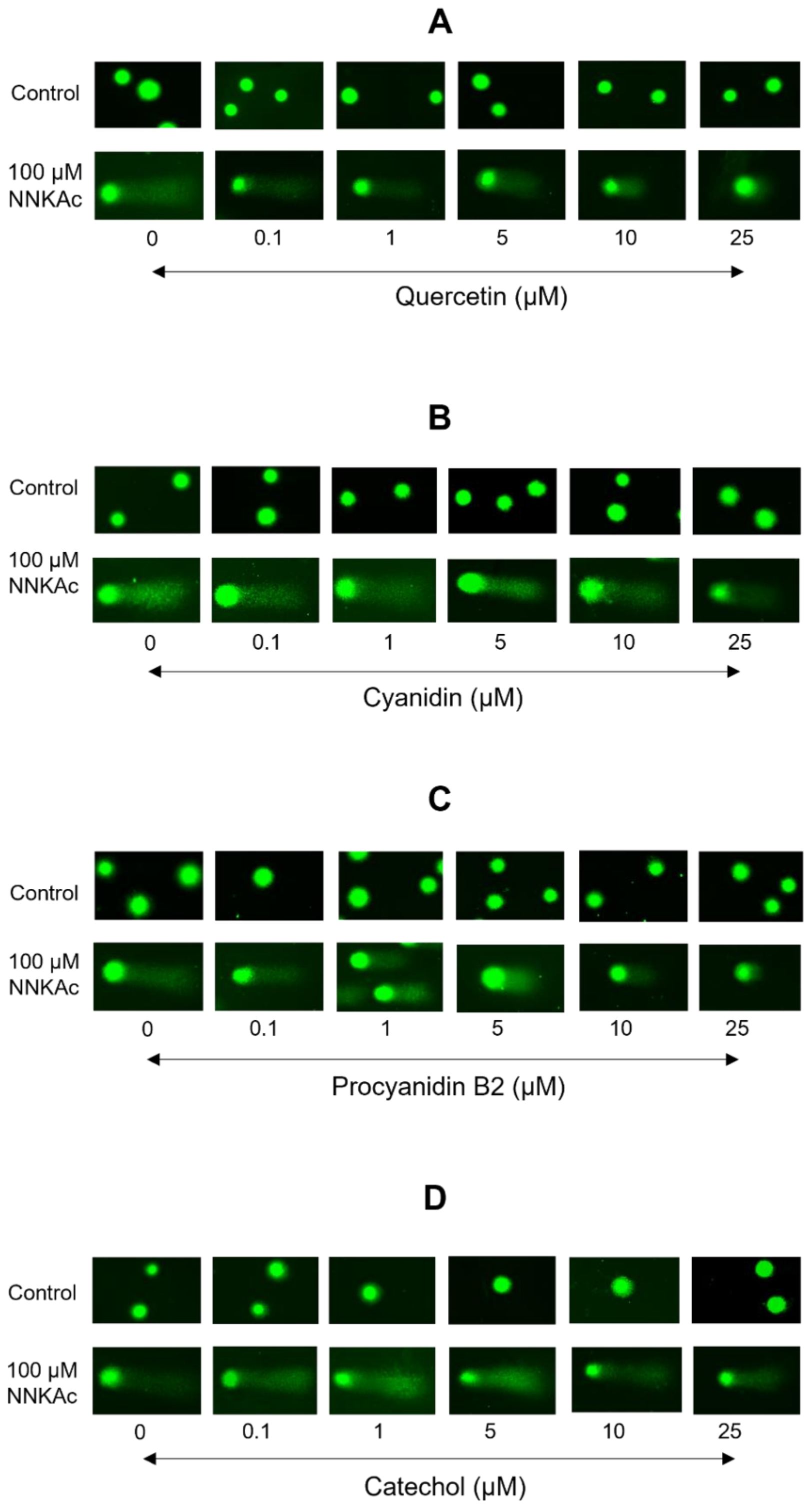

Figure S7: Effect of quercetin (A), cyanidin (B), procyanidin B2 (C), and catechol (D) on NNKAc-induced DNA damage in BEAS-2B cells measured by comet assay. Abbreviations: NNKAc: 4-[(acetoxymethyl)nitrosamino]-1-(3-pyridyl)-1-butanone.

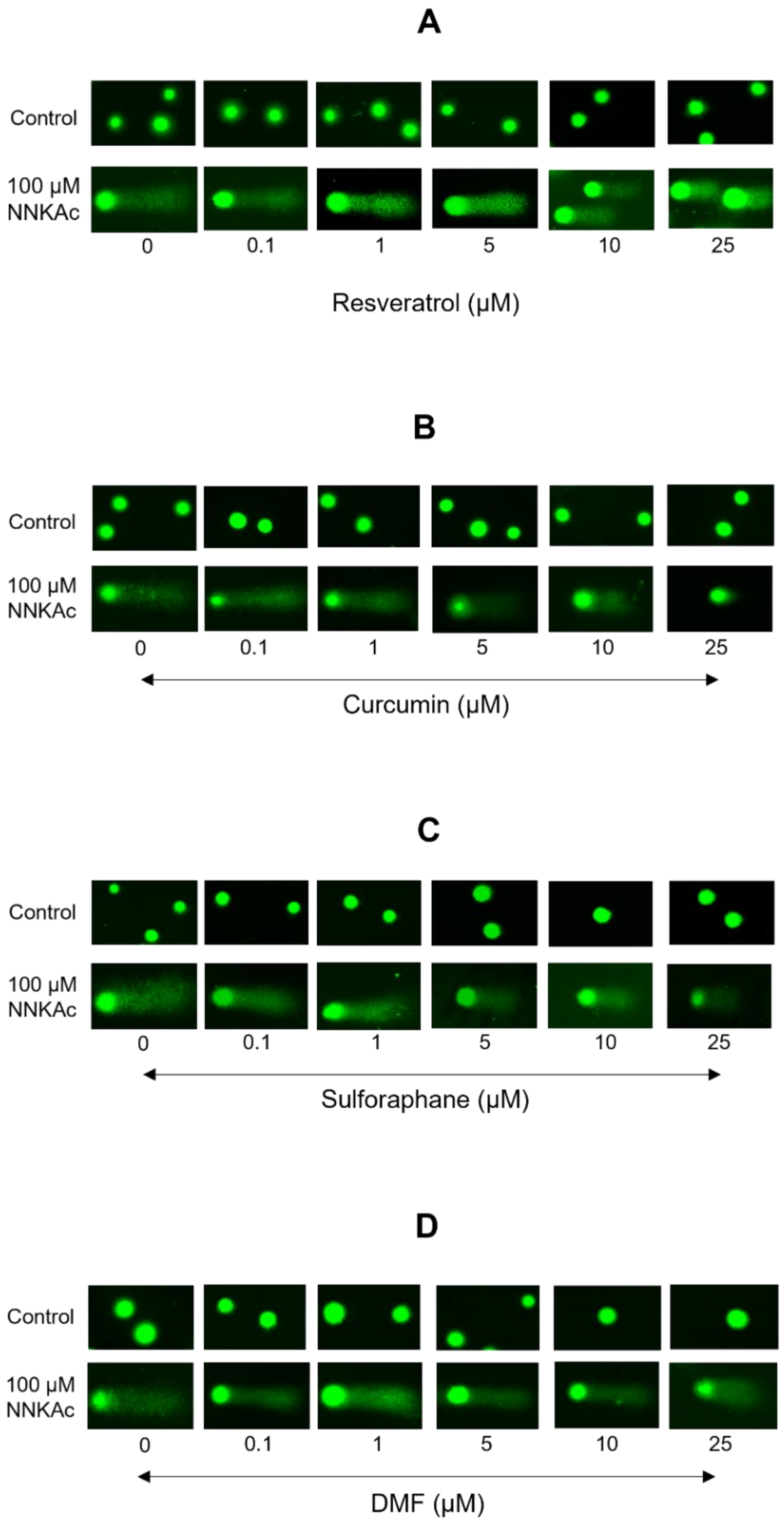

Figure S8: Effect of resveratrol (A), curcumin (B), sulforaphane (C), and DMF (D) on NNKAc-induced DNA damage in BEAS-2B cells measured by comet assay. Abbreviations: NNKAc: 4-[(acetoxymethyl)nitrosamino]-1-(3-pyridyl)-1-butanone.
